# HSP27 functional switch drives castration-resistant prostate cancer *via* mTOR pathway activation, highlighting promising combination therapies

**DOI:** 10.1186/s13046-026-03695-6

**Published:** 2026-03-25

**Authors:** Quang Hieu Duong, Thi Khanh Le, Thi Thom Tran, Stéphane Audebert, Luc Camoin, Ngan Giang Nguyen, Michael Baboudjian, Sophie Giusiano, Uyen Thi Tu Phan, Souhaila Zaghdoudi, José Adelaide, Virginie Baylot, Martin Gleave, Carmen Garrido, David Taïeb, Palma Rocchi

**Affiliations:** 1 Aix Marseille Univ, CNRS, CINAM, ERL INSERM U 1326, CERIMED, Marseille, France; 2 INSERM UMR 1231, HSP-Pathies, Centre for Translational and Molecular Medicine, University of Burgundy Europe, Dijon, 21000 France; 3 Centre Georges-François Leclerc (CGFL), Dijon, 21000 France; 4 Marseille Protéomique, Centre de Recherche en Cancérologie de Marseille, INSERM, CNRS, Institut Paoli-Calmettes, Aix-Marseille University, Marseille, 13009 France; 5 Institute of Molecular Biology, Academia Sinica, Nangang, Taipei City, 115014 Taiwan; 6 Laboratoire d’Anatomie Et Cytologie Pathologiques Hôpital Timone, Marseille, 13005 France; 7 Predictive Oncology Laboratory, Centre de Recherche en Cancérologie de Marseille, Inserm UMR 1068, CNRS UMR 7258, Institut Paoli-Calmettes, Aix-Marseille University, 27 Bd. Leï Roure, Marseille, 13273 France; 8 The Vancouver Prostate Centre, University of British Columbia, Vancouver, BC V6H 3Z6 Canada; 9 Department of Urology, North hospital , APHM-AMU, Marseille, 13015 France; 10 Department of Nuclear Medicine, APHM-AMU, Marseille, 13005 France

**Keywords:** HSP27, MTOR, Castration-resistant prostate cancer (CRPC), OGX-427 (Apatorsen), Sapanisertib, Interactome, Patient-derived organoids (PDOs)

## Abstract

**Background:**

Castration-resistant prostate cancer (CRPC) is an advanced and ultimately incurable stage of the disease that arises despite androgen deprivation and remains challenging to treat due to the limited and short-lived efficacy of current therapies. Heat shock protein 27 (HSP27), a molecular chaperone, has been implicated in prostate cancer (PC) progression and therapy resistance; however, its mechanistic roles remain incompletely understood. This study aimed to delineate the HSP27 interactome in PC cells during PC progression and to explore its functional significance, particularly in relation to the mTOR signaling pathway, which is activated in most cases, primarily due to PTEN loss.

**Methods:**

We performed affinity purification-mass spectrometry (AP-MS) to identify HSP27-interacting proteins in a panel of prostate cell lines with increasing aggressiveness: PNT1A (non-malignant), LNCaP (androgen-sensitive), DU-145, and PC-3 (both androgen-independent CRPC models). Functional enrichment and network analyses were conducted to uncover pathways associated with HSP27 interactors. Validation experiments included Western blotting, co-immunoprecipitation, and pharmacological inhibition using OGX-427 and mTOR inhibitors (Everolimus, Sapanisertib) in prostate cancer cell lines, patient-derived organoids (PDOs), and xenograft models.

**Results:**

HSP27 exhibited a progressively expanded interactome in CRPC models, with enrichment of proteins involved in stress adaptation, proteostasis, and mTOR signaling. These insights highlight HSP27 not only as a molecular chaperone but also as a dynamic network hub that may promotes survival in stress-adapted tumor states. In PC-3 cells, HSP27 stabilized key mTORC1 components, including RAPTOR, S6K1, and 4E-BP1 via its chaperone function, thereby enhancing mTORC1 activation. Combined inhibition of HSP27 using the antisense oligonucleotide OGX-427 (Apatorsen) and mTOR blockade via Sapanisertib induced a robust synergistic anti-tumor effect across diverse preclinical models, including advanced PC Patient-derived organoid (PDOs) and CRPC xenografts.

**Conclusions:**

Our findings reveal novel insights into HSP27’s role in PC progression and its modulation of the mTOR signaling pathway in CRPC, highlighting dual HSP27/mTOR inhibition as a promising therapeutic approach for advanced, treatment-resistant disease.

**Graphical Abstract:**

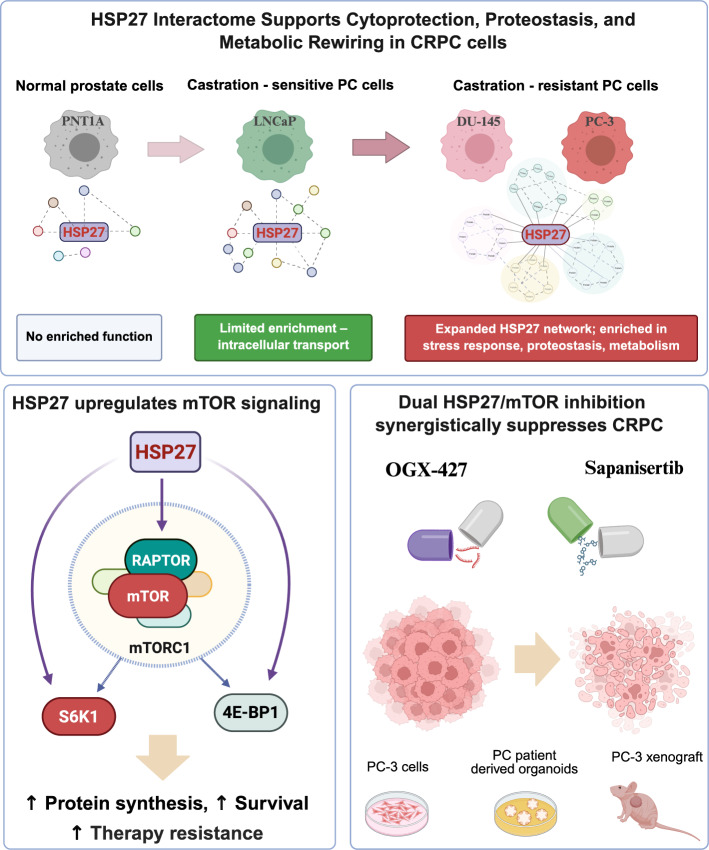

**Supplementary Information:**

The online version contains supplementary material available at 10.1186/s13046-026-03695-6.

## Background

Prostate cancer (PC) is the second most common cancer and the fifth leading cause of cancer death among men [1, 2]. At advanced stages, patients are initially treated with doublet (Docetaxel + androgen deprivation therapy/ADT) or triplet therapy (Docetaxel + ADT + androgen-receptor pathway inhibitor), the choice of therapy being influenced by the timing of metastasis after primary treatment (metachronous vs synchronous) and tumor volumes [3–6]. However, the disease inevitably progresses to a castration-resistant PC phenotype (CRPC) due to various mechanisms that enable tumor growth despite low androgen levels. These mechanisms involve both AR-dependent pathways, such as AR overexpression, AR mutations, and increased steroidogenic signalling pathways, and non-AR-related pathways. Treatment options for CRPC include chemotherapy, PARP inhibitors for BRCA and ATM-mutated cases, and PSMA-targeted radionuclide therapy; however, these therapies often demonstrate limited duration of efficacy [7].

We previously described Heat Shock Protein 27 (HSP27), also called Heat Shock Protein Family B (Small) Member 1 (HSPB1) as an important driver in CRPC progression. Depletion of HSP27 expression using OGX-427 (also known as Apatorsen, a second-generation antisense oligonucleotide targeting HSP27 mRNA) promoted apoptosis and restored therapy sensitivity to hormone suppression and taxanes chemotherapy in both in vivo and in vitro models [8–10]. The anti-cancer activity of HSP27 inhibition has been reported in a randomized clinical trial phase II where PSA response rates were doubled in CRPC patients co-treated with OGX-427 compared to prednisone alone (NCT01120470) [11].

From a mechanistic standpoint, our laboratory has demonstrated that HSP27 promotes CRPC progression through its cytoprotective chaperone activity, which facilitates the stabilization and upregulation of oncogenic client proteins, including TCTP, eIF4E, Menin, and DDX5 [12–15]. Using yeast two-hybrid screening with human cDNA libraries derived from HeLa cancer cells and normal testis tissue, we identified a number of novel HSP27-interacting partners and uncovered previously unrecognized functions of HSP27, including roles in alternative splicing and DNA repair [16]. Notably, many of these functions were specific to the cancer-derived library, underscoring HSP27’s involvement in tumor-specific regulatory pathways.

Despite these insights, the molecular mechanisms by which HSP27 promotes CRPC progression remain incompletely understood. In the present study, we sought to further elucidate the HSP27 protein–protein interaction (PPI) network in prostate cancer by identifying and charactering its interactome across four human prostate cell lines representing distinct stages of tumor progression: PNT1A, LNCaP, DU-145, and PC-3. Our analysis revealed that HSP27 may progressively expand its protein–protein interaction (PPI) landscape during prostate cancer evolution, and its interactome may play a central role in supporting stress adaptation, proteostasis, and metabolic plasticity particularly in CRPC cell lines. We also show that HSP27 acts as a key regulator of mTORC1 signaling in CRPC cells. Notably, we demonstrate that co-targeting HSP27 with OGX-427 and mTOR with Sapanisertib elicited a robust synergistic anti-tumor response in CRPC cell lines and patient-derived organoids, and significantly reduced tumor growth in a CRPC xenograft model. Collectively, these results not only underscore the central role of HSP27 in driving PC progression but also highlight a promising combinatorial therapeutic strategy for overcoming treatment resistance in CRPC.

## Methods

### Cell lines and cell culture methods

In this project, four different types of human prostatic cell lines were used: normal prostatic cells (PNT1A), castration-sensitive prostate cancer (CSPC) cells (LNCaP), castration-resistant prostate cancer (CRPC) cells (DU-145 and PC-3) to investigate the roles of HSP27 during the progression of CRPC. PNT1A and LNCaP cells were cultivated in RPMI-1640 medium (Roswell Park Memorial Institute),while DU-145 and PC-3 were maintained in Dulbecco’s Modified Eagle’s Medium (DMEM). All media were supplemented with 10% of fetal bovine serum (FBS), and cells were incubated at 37 °C in a humidified atmosphere containing 5% CO_2_.

### Affinity purification coupled to mass spectrometry (AP/MS)

The AP/MS procedure has been previously outlined [17]. The protein extracts from the 4 cell lines (PNT1A, LNCaP, DU-145, and PC-3) were pre-cleaned with 40 μl of the protein A Sepharose (Protein A Sepharose® 4 Fast Flow, REF.17–5280-01, GE Healthcare, MERCK), and were incubated with 5 μg of Ab against HSP27 (Supplementary Material-2 Table S1) overnight at 4 °C. Subsequently, the immunoprecipitated complexes were captured by incubating with 40 μl of protein A Sepharose bead for 1 h, 4 °C, which was followed by 3 times of washing using the lysis buffer. Ultimately, the resulting beads were suspended with 20 μl Laemmli sample buffer 4X, and heated at 95 °C for 5 min. To evaluate the efficiency of the IP, 10% of the samples were run on the SDS-PAGE for silver staining analysis as previously described [18].

The IP samples were analyzed by liquid chromatography (LC)-tandem MS (LC-MSMS) using a Q Exactive Plus Hybrid Quadrupole-Orbitrap online with a nanoLC Ultimate 3000 chromatography system (Thermo Fisher Scientific™, San Jose, CA). Concerning data processing protocol, relative intensity-based label-free quantification (LFQ) was processed using the MaxLFQ [19] algorithm from the freely available MaxQuant computational proteomics platform, version 1.6.2.1 [20]. Analysis was done on biological triplicates, each injected three times on mass spectrometers.

### Kaplan–Meier survival analysis

The data cohort was derived from the West Coast Prostate Cancer Dream Team project, Stand Up 2 Cancer and the Prostate Cancer Foundation. The samples in this study were collected from men with metastatic castration-resistant prostate cancer (mCRPC) [21]. RNA sequencing data and clinical data are available for 96 out of 101 tumor biopsies. Patients were stratified into high and low expression groups based on the gene expression levels in (logTPM + 1) data (TPM: transcripts per million). A patient was assigned to the high expression group if their (logTPM + 1) value was greater than or equal to the median; otherwise, they were placed in the low expression group. Survival analyses were restricted to 24 months, as the number of patients at risk decreased markedly beyond this time point, limiting statistical power. Among several tested intervals, the 24-month cutoff yielded the most consistent and significant survival differences between expression groups. Hazard ratios (HRs) were estimated using Cox proportional hazards regression models, and statistical significance between survival curves was assessed using the log-rank test, with a *P*-value < 0.05 indicating a statistically significant difference. The analysis was conducted using the survminer package (version 0.5.0) and survival (version 3.7–0) in R 4.2.2 (https://cran.r-project.org/).

### ASO and siRNA transfection

Cells at about 50% confluence were transfected twice with ASOs or siRNAs at the indicated concentrations. The ASOs or siRNAs were pre-incubated with 1 mL of Gibco Opti-MEM (Life Technologies, Courtaboeuf, France) containing 4 µL of Oligofectamine (Life Technologies, Courtaboeuf, France) for 20–30 min prior to addition to the cells. After 4 h of incubation, the mixture was removed and replaced with fresh complete medium. The same treatment was repeated the next day. Seventy-two hours after the first transfection, cells were used for MTT assays or harvested for downstream experiments such as Western Blotting (WB) or RT-qPCR. The ASO control or siControl containing scrambled sequences (SCRs), which do not target any known gene, were used as negative controls under the same experimental conditions.

### Protein extraction, quantification, and Western blot analysis

Transfected cells with OGX-427 and scrambled control after harvesting were washed by Dulbecco’s phosphate buffered saline 1X (DPBS, Life Technologies, Courtaboeuf, France), and then were lysed by lysis buffer supplemented with 1x protease inhibitor cocktail (Roche) and 1x phosphatase inhibitor Na_3_VO_4_ (Sigma-Aldrich) for 30 min on ice. Protein lysates were collected by centrifugation at 4 °C, 13,400 rpm for 40 min (collecting supernatant), then were quantified following the BicinChoninic acid protein assay kit (BCA, Pierce™, Life Technologies, Courtaboeuf, France), with absorbance measured at 540 nm using spectrophotometer (Tecan Sunrise).

Protein expression levels were analyzed by Western blotting. Based on BCA quantification, 30 μg of total protein per sample was mixed with sample buffer (Invitrogen) and denatured at 95 °C for 10 min prior to loading onto 4–12% SDS-PAGE gels (Life Technologies, Courtaboeuf, France). Electrophoresis was performed at 90 V for 10 min, followed by 120 V for 90 min in 1x NuPAGE MOPS SDS running buffer (Life Technologies, Courtaboeuf, France). Proteins were then transferred from the SDS-PAGE gel onto Nitrocellulose membrane in 1x transfer buffer (192 mM Glycine, 25 mM Tris, 1% SDS) supplemented with 20% ethanol (Carlo ERBA Reagents SAS, 27,106 Val de Reuil, France) overnight at 28V. The membranes were blocked with 5% bovine serum albumin (BSA) in 1x Tris-buffered saline (TBS, Euromedex, 67,460 Souffelweyersheim, France) containing 0.1% of Tween-20 (Life Technologies, Courtaboeuf, France). To specifically detect target proteins, nitrocellulose membranes were incubated overnight at 4 °C with primary antibodies as detailed in Supplementary Material 2- Table S1. Following incubation, membranes were washed 3 times (30 min in total), and then incubated with horseradish peroxidase (HRP)-conjugated secondary antibodies (anti-rabbit or anti-mouse, Santa Cruz Biotechnology, CA, USA) diluted in BSA 5% for 90 min at room temperature. Protein signals were visualized using ELC prime western blot detection reagent (GE Healthcare).

### RNA extraction and quantitative Real-Time PCR (RT- qPCR)

Total RNA was extracted using the RNeasy Mini Kit’s manufacturer protocol (QIAGEN®). Complementary DNA (cDNA) synthesis was carried out using the SuperScript™ VILO™ cDNA Synthesis Kit (Invitrogen™, Thermo Fisher Scientific, Life Technologies SAS, Villebon-sur-Yvette, France). Quantitative PCR (qPCR) were performed using the TaqMan™ Universal PCR Master Mix (Applied Biosystems™, Thermo Fisher Scientific, Life Technologies SAS, Villebon-sur-Yvette, France), on a CFX96 Touch Real-Time PCR Detection System (Bio-Rad Laboratories, USA). The expression level of the GAPDH gene was used as an internal control. The GAPDH primers and probe were obtained from the product namely The Human GAPD (GAPDH) Endogenous Control (FAM™/MGB probe, non-primer limited) (Applied Biosystems™, Thermo Fisher Scientific, Life Technologies SAS, Villebon-sur-Yvette, France). Primer and probe sequences used to analyze mRNA levels of HSP27, S6K1, 4E-BP1, RAPTOR, were listed in the Supplementary material 2- Table S2. Each sample was analysed in triplicate, and relative gene expression was calculated using the 2^−ΔΔCT^ method [22].

### Culture and characterization of patient-derived organoids (PDOs) 

We are currently developing different preclinical models using tumors and biopsies from CSPC and CRPC patients in our laboratory (Tran T.T., submitted manuscript). These samples were collected following AP-HM Biological Resources Center Authorization (#AC-2023–5572). Prostate resections are performed by Dr. Michael Baboudjian (AP-HM urologist Assistant). PC organoids are cultured in droplets embedded in Matrigel (Corning® Matrigel® Growth Factor Reduced (GFR) Basement Membrane Matrix, Sigma Aldrich Chimie S.a.r.l, 38,297 Saint-Quentin-Fallavier Cedex France) supplemented with a cocktail of growth factors and serum-free medium following the standard protocol from the study Drost J et al. [23]. PDOs and their original tumors were fixed in 4% formalin overnight at 4 °C, then were dehydrated in ethanol, embedded into paraffin then were sectioned at 4 µm. Specimens were then characterized by Hematoxylin and Eosin staining, Immunohistochemistry and Immunofluorescence to assess their recapitulation features including morphology, expression of prostate cancer markers (AR, PSMA, CK5/CK8, AMACR) and to evaluate the expression of HSP27 and phosphorylated mTOR (S2448) in comparison with their original tumor tissues from PC patients, following standard protocol described [24]. Organoids used in this study were established from tumor samples of castration-sensitive prostate cancer (CSPC) patients classified as high-risk (Patients #11, #19, and #24) or unfavorable intermediate-risk (Patient #23).

### Combination Index (CI) analysis of drug combinations and cell viability assay

In order to check if the combinations of OGX-427 with Everolimus or Sapanisertib can induce synergistic anti-cancer activity in PC-3 cells, we realized a combination index (CI) analysis based on the median effect of the combined therapies compared with the activity of each treatment in monotherapy. The CI values were calculated following the Chou and Talalay method [25] using CompuSyn software (ComboSyn Inc., Paramus, NJ, USA). CI values were encoded as recommended by Chou and Talalay [25]. + + + + + Very strong synergism CI < 0.1; + + + + strong synergism CI 0.1–0.3; + + + synergism CI 0.3–0.7; + + moderate synergism CI 0.7–0.85; + slight synergism CI 0.85–0.90; nearly additive CI 0.9–1.10; CI values > 1.1 were considered as antagonism. 

In the monotherapy experiments, OGX-427 was tested at concentrations from 50 to 300 nM, while Everolimus and Sepanisertib ranged from 10 to 4000 nM. MTT assays were performed 3 days after treatment.

Drug combination ratios were established based on in vitro potency and mechanistic rationale. OGX-427 showed approximately two-fold higher potency (lower IC₅₀) than Sapanisertib, supporting a 2:1 ratio (OGX-427: Sapanisertib) to achieve near-equipotent exposure and balanced pharmacodynamic effects. Everolimus displayed an IC₅₀ about ten-fold higher than OGX-427, with detectable activity only above ~ 100 nM. Applying a proportional 10:1 ratio would therefore require supra-physiological OGX-427 concentrations (~ 1000 nM), increasing the risk of off-target effects. Mechanistically, HSP27 regulates the mTOR pathway at multiple levels; thus, robust HSP27 inhibition by OGX-427 is expected to potentiate mTOR pathway suppression by either sapanisertib or everolimus. Accordingly, a 2:1 ratio was selected as a mechanistically justified and experimentally feasible condition, ensuring potent HSP27 inhibition alongside biologically relevant suppression of the mTOR pathway.

For the combination therapy, a constant 2:1 ratio of OGX-427 to Everolimus or Sepanisertib was maintained, with OGX-427 concentrations between 50 and 300 nM. After 24 h of OGX-427 treatment, Everolimus or Sepanisertib were added, and cell viability assays were carried out 2 days later.

In this study, cell viability assays for 2D cell line cultures were performed using the PrestoBlue™ Cell Viability Reagent (Invitrogen™, Thermo Fisher Scientific, Life Technologies SAS, Villebon-sur-Yvette, France), while the CellTiter-Glo® 3D Cell Viability Assay (Promega, Charbonnières-les-Bains, France) was employed to assess the viability of the 3D organoid model.

### Preclinical evaluation of drug combinations in PC-3 xenografts

PC-3 cells (5 × 10^6^) in 100 μL of 1x DPBS were inoculated subcutaneously into the right flank of 6-week-old male NMRI nude mice (Envigo RMS S.A.R.L., 92,777 Paris La Defense, France). Treatment began when tumors reached a size of 50 to 100 mm^3^. The mice were randomly divided into 5 treatment groups: DMSO 2%, SCR ASO, OGX-427, Sepanisertib, and a combination of OGX-427 and Sepanisertib. The ASOs were administered intraperitoneally (IP) on weekdays (Monday to Friday) at a dose of 10 mg/kg while Sepanisertib was given intraperitoneally 3 times per week at 5 mg/kg. Tumor volume (mm^3^) was measured weekly using a calliper in three perpendicular dimensions (X = width, Y = length, Z = depth), and calculated following the formula X × Y × Z × 0.5236. Throughout the treatment period, the mice were monitored for signs of systemic toxicity, and their body weights were measured daily. The mice were housed in the animal facility of the Centre Européen de Recherche en Imagerie Médicale, CERIMED (agreement number D1305532). P.R. owns a personal agreement (#A13-477) for the animal handling and experimentation for this study. All animal experiments were conducted in accordance with ethical guidelines.

### Cell cycle assays

To evaluate the impact of the OGX-427 and Sapanisertib combination on the cell cycle, PC-3 cells (50,000) were seeded into 6-well plates and allowed to adhere for one day before treatment. Cells were exposed to either OGX-427 (200 nM), Sapanisertib (400 nM), or a combination of both. DMSO and Scramble (SCR ASO) served as vehicle controls. After 48 h of treatment, cells were harvested, washed with 1x DPBS, and fixed in 70% cold ethanol for one hour. The nuclei were stained with propidium iodide (P3566, Life Technologies, Courtaboeuf, France) according to the manufacturer’s instructions. The cell cycle was analyzed by flow cytometry using CytoflexS (Beckman Coulter, Inc.) with a minimum of 5,000 events per sample.

### Immunofluorescence staining for Ki-67

Tumor xenografts were harvested and fixed in 10% paraformaldehyde at room temperature, then were embedded in paraffin, and cut into different specimens (4 µm). The tissue specimens were then deparaffined and stained with Ki-67 polyclonal rabbit antibody (proliferation marker, 27,309–1-AP, Proteintech), then was incubated with secondary antibody conjugated with Alexa Fluor 488 (A32731, Thermo Fisher Scientific, Life Technologies SAS, Villebon-sur-Yvette, France) and DAPI for nucleus staining (Thermo Fisher Scientific, Life Technologies SAS, Villebon-sur-Yvette, France) following the manufacturer's instruction. Ki-67-positive nucleus was detected by Apotome microscopy (Zeiss Axio Imager Z2).

### Quantification of protein signals and statistical analysis

Protein band intensities from Western blot analyses were quantified by ImageJ software (https://imagej.nih.gov/ij/). Glyceraldehyde 3-phosphate dehydrogenase (GAPDH), VINCULIN were used as loading controls to normalize protein expression levels across samples. Statistical analyses were performed using GraphPad Prism 10 software (GraphPad Software, Inc). The specific statistical tests used for each experiment are indicated in the corresponding figure legends. 

## Results

### Functional switch of HSP27 during PC progression

**Fig. 1:**
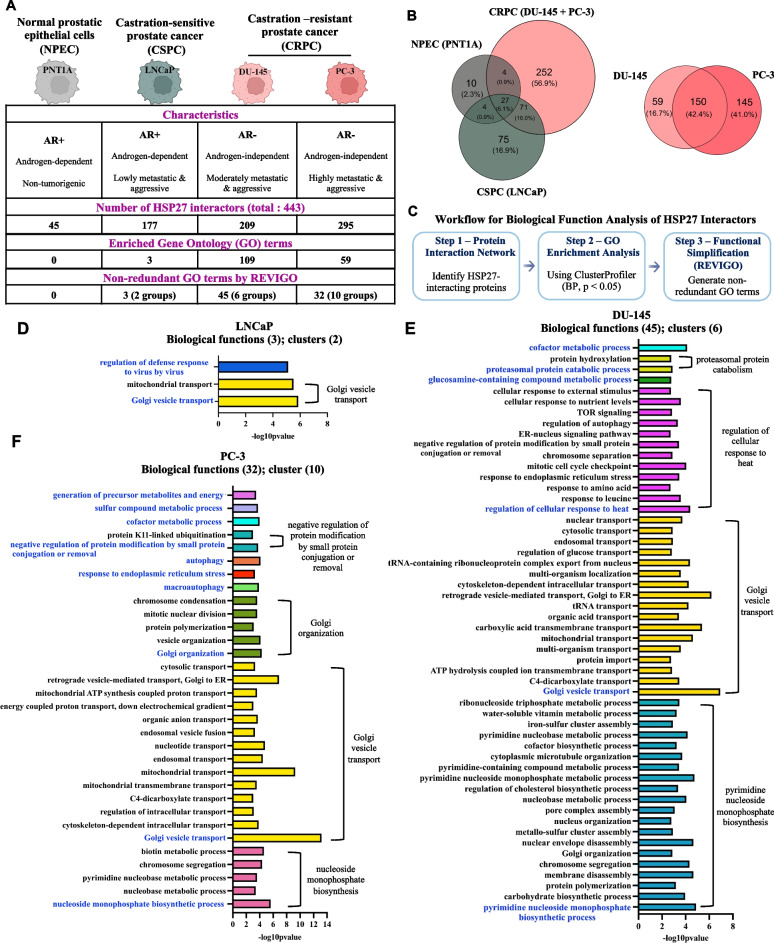
Progressive expansion and functional rewiring of the HSP27 interactome during prostate cancer progression. **A** We performed affinity purification coupled with mass spectrometry (AP-MS) across a panel of human prostatic cell lines representing increasing levels of aggressiveness and metastatic potential: normal prostatic epithelial cells (PNT1A; non-malignant control), castration-sensitive prostate cancer (CSPC) cells (LNCaP; androgen-dependent, low metastatic and low aggressive potential), and castration-resistant prostate cancer (CRPC) cells (DU145; moderately metastatic/aggressive and PC-3; highly metastatic/aggressive). We identified a total of 443 HSP27-associated proteins across the four cell lines. The number of HSP27 interactors increased with disease progression. **B** The Venn diagram displayed the overlapping proteins and the uncommon ones among 4 cell lines, including normal prostatic cells (PNT1A) vs (CSPC cells (LNCaP) vs CRPC( cells (DU-145 and PC-3) (left panel); and between DU-145 vs PC-3 (right panel). **C** Gene Ontology (GO) enrichment analysis (Biological Process category) of HSP27 interactors was performed using ClusterProfiler (*p* < 0.05, Benjamini–Hochberg corrected) and summarized using REVIGO to reduce redundancy. **D-F** Bar charts showing biological processes associated with the HSP27 interactome in LNCaP (**D**), DU-145 (**E**), and PC-3 (**F**), as determined by REVIGO analysis. The x-axis represents –log₁₀ (*adjusted p*-value) indicating enrichment significance. Each bar corresponds to a specific GO term (Biological Process category). Each cluster represents a set of related biological processes and is visualized as color-coded groups of bars in the charts. To annotate the functional groups identified by REVIGO, we selected the most significantly enriched Gene Ontology (GO) biological process—defined by the lowest p-value—as the representative label for each group (indicated in blue). In LNCaP cell, there is only two functional groups or clusters including Golgi vesicle transport and regulation of defense response to virus by virus (**D**). In DU-145 cell, the REVIGO identify 45 functions which are ultimately categorized into 6 groups of functions including: pyrimidine nucleoside monophosphate biosynthesis; Golgi vesicle transport; regulation of cellular response to heat; glucosamine-containing compound metabolism; proteasomal protein catabolism; cofactor metabolism (**E**). In PC-3 cell, there are 32 representative terms joining into 10 different functional groups including Golgi vesicle transport, nucleoside monophosphate biosynthetic process, Golgi organization, and negative regulation of protein modification by small protein conjugation or removal, autophagy, cofactor metabolic process, macro-autophagy, sulfur compound metabolic process, generation of precursor metabolites and energy and response to endoplasmic reticulum stress (**F**).

To elucidate the role of HSP27 in PC progression, we performed affinity purification coupled with mass spectrometry (AP-MS) on a panel of human prostatic cell lines exhibiting progressively increasing aggressive and metastatic potential, recapitulating various stages of disease progression (Fig. 1A). This panel included normal prostatic epithelial cells (PNT1A: a non-malignant control), castration-sensitive prostate cancer (CSPC) cells (LNCaP: androgen-dependent, low metastatic and aggressive potential), and castration-resistant prostate cancer (CRPC) cells (DU-145: moderately metastatic/aggressive and PC-3: highly metastatic and aggressive). These two CRPC cell lines lack androgen receptor (AR) and prostate-specific antigen (PSA) expression [26].

Across the four cell lines, a total of 443 HSP27-interacting proteins were identified. Importantly, we observed a progressive increase in the number of HSP27 interactors correlating with prostate cancer advancement (Fig. 1A, Supplementary Material 1 ). The Venn diagram shows 27 interactors shared among the 4 cell lines. LNCaP shared 98 interactors with the CRPC cell lines, accounting for about 55% of LNCaP's HSP27-interacting proteins (Fig. 1B, left panel). The two CRPC lines shared up to 150 HSP27-interaction partners, which contribute to nearly 72% of HSP27 interactors in DU-145 cells and approximately 50% in PC-3 (Fig. 1B, right panel). These observations suggest that an important number of HSP27 functions are shared in the two CRPC models.

To gain functional insights, we performed Gene Ontology (GO) enrichment analysis of the HSP27- interacting proteins in each cell line using the ClusterProfiler package implemented in R [22], focusing on the Biological Process (BP) category (*p*-values < 0.05). The enriched GO terms were further refined using REVIGO to reduce redundancy and highlight representative biological functions (accessed on 28th October, 2020, default settings) [27] (Fig. 1C). No significant enrichment was observed for the HSP27-interacting proteins in the normal prostate epithelial cell line PNT1A. In contrast, the androgen-sensitive LNCaP cell line showed limited enrichment, with only three GO terms, two of which were related to intracellular transport (Golgi vesicle transport and mitochondrial transport) (Fig. 1D). Notably, the CRPC cell lines DU-145 and PC-3 exhibited extensive enrichment in biological processes, with 109 and 59 GO terms, respectively (Supplementary Material 2-Figure S1, Supplementary Material 3). REVIGO summarized these into 45 biological functions grouped into six clusters for DU-145 (Fig. 1E) and 32 functions across ten clusters for PC-3 (Fig. 1F). Each cluster represents a set of related biological processes and is visualized as color-coded groups of bars in the charts.

Together, these findings demonstrate a potential functional switch of HSP27 during PC progression, with a CRPC-specific gain of functions. This network rewiring suggests that HSP27 acquires additional context-specific roles, likely contributing to cellular adaptation, enhanced survival, and drug resistance mechanisms in aggressive, AR-independent prostate cancer.

### HSP27 interactome supports cytoprotection, proteostasis, and metabolic rewiring in CRPC cells

Enrichment analysis revealed that stress response pathways were selectively overrepresented in the HSP27 interaction networks of CRPC cell lines DU-145 and PC-3. These included **“**response to endoplasmic reticulum (ER) stress**”** (in both DU-145 and PC-3)**, “**macroautophagy”, “autophagy**”** (PC-3), as well as the biological functions in the cluster **“**regulation of cellular response to heat” in DU-145 such as cellular response to external stimulus, mTOR pathway, mitotic cell cycle checkpoint, etc. (Fig. 1E & F). These pathways are hallmark indicated of cytoprotective responses, consistent with the established role of HSP27 in mitigating proteotoxic and environmental stress, particularly in therapy-resistant contexts [28, 29].

Beyond stress responses, the HSP27 interactome in CRPC cells was significantly enriched in proteostasis-associated pathways, such as “proteasomal protein catabolic process” in DU-145 (Fig. 1E) and “negative regulation of protein modification by small protein conjugation or removal” (DU-145 and PC-3, Figure 1E & F). These results suggest that HSP27 may coordinate enhanced protein quality control mechanisms in CRPC cells through its protein interaction network. Additionally, metabolic flexibility was evident through the enrichment of nucleotide biosynthesis (e.g., pyrimidine nucleoside monophosphate biosynthesis) and a broad spectrum of metabolic processes: cofactor metabolism and glucosamine-containing compound metabolism in DU-145; sulfur compound metabolism and generation of precursor metabolites and energy in PC-3.

Together, these findings indicate that the HSP27 interactome may play a central role in supporting stress adaptation, proteostasis, and metabolic plasticity specifically in CRPC cell lines, in line with their aggressive and drug-resistant phenotypes. In contrast, LNCaP displays a narrower HSP27-associated biological functions, consistent with its androgen-dependent, less aggressive nature. Among the pathways specifically enriched in CRPC cells, we focused on the mTOR signaling cascade, a critical regulator of cell growth and survival, due to its known roles in PC progression and therapy resistance. The potential role of HSP27 in modulating this pathway in PC remains underexplored and is therefore studied in the subsequent sections of this work.

### HSP27 promotes mTORC1 signaling in CRPC through stabilization of RAPTOR, 4E-BP1, and S6K1

**Fig. 2:**
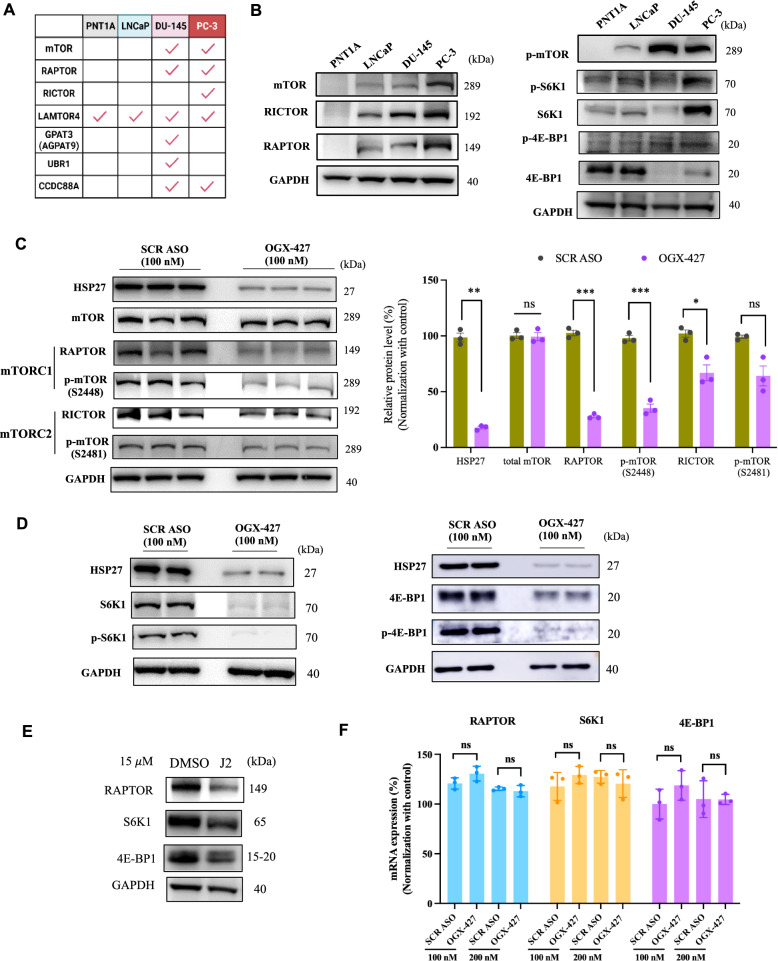
HSP27 Promotes mTORC1 Signaling in CRPC Through Stabilization of RAPTOR, 4E-BP1, and S6K1 in CRPC PC-3 cells. **A** HSP27-interacting proteins involving in mTOR pathway were identified in each cell line by AP/MS. **B** Western blot analysis showing higher expression levels of mTOR, RAPTOR, and RICTOR in CRPC cell lines (DU-145, PC-3) compared to CSPC LNCaP cells, consistent with elevated mTORC1 activity in CRPC models. **C** PC-3 cells were treated with the HSP27 antisense oligonucleotide OGX-427 to silence HSP27. Immunoblotting revealed a marked reduction in phosphorylated mTOR at Ser2448 (p-mTOR S2448, a marker of activated mTORC1) and RAPTOR expression, while phosphorylated mTOR at Ser2481 (mTORC2-specific) and RICTOR were only modestly reduced. Total mTOR levels remained unchanged, suggesting that HSP27 selectively regulates mTORC1 activity. **D** HSP27 silencing also led to decreased expression of mTORC1 downstream effectors, including 4E-BP1, phospho-4E-BP1 (p-4E-BP1), S6K1, and phospho-S6K1 (p-S6K1). **E** Similar effects were observed upon treatment with J2, a small-molecule inhibitor of HSP27 that disrupts its oligomeric state and chaperone function. J2 treatment reduced protein levels of RAPTOR, 4E-BP1, and S6K1. **F** RT-qPCR analysis of the corresponding mRNAs (RAPTOR, 4E-BP1, S6K1) showed no significant changes in transcript levels upon HSP27 inhibition, indicating that HSP27 stabilizes these proteins post-transcriptionally. The experiment was performed in triplicate, and the blots shown in the manuscript represent one of the biological replicates. Statistical analysis was performed using an unpaired two-tailed Student’s t-test to compare two groups. Data are presented as mean ± SEM (Standard Error of Mean). Results with *P* < 0.05 were considered statistically significant and are indicated by *, *P* < 0.05; **, *P* < 0.01; ***, *P* < 0.001, and ****, *P* < 0.0001, ns: non-significant

Proteomic analysis revealed that HSP27 interacts with the core subunit proteins of the mTOR complexes (mTORC) in CRPC models, including mTOR (shared by mTORC1 and mTORC2), RAPTOR (specific regulatory protein of mTORC1), and RICTOR (specific regulatory subunit of mTORC2) (Fig. 2A). Notably, all three proteins exhibited elevated expression in DU-145 and PC-3 cells compared to LNCaP, with a concomitant increase in mTORC1 activity (Fig. 2B, Supplementary Material 2- Figure S2A).

To investigate whether HSP27 modulates mTOR pathway activity, we analyzed the effects of HSP27 inhibition in PC-3 cells. Silencing HSP27 via OGX-427 treatment had no effect on total mTOR level, but significantly reduced phosphorylated mTOR at Ser2448 (p-mTOR S2448, specific for activated mTORC1) and RAPTOR expression. In contrast, phosphorylated mTOR at Ser2481 (mTORC2-specific) and RICTOR levels were only modestly reduced (Fig. 2C), indicating selective modulation of mTORC1 by HSP27. These findings suggest that HSP27 more strongly influences mTORC1 activity.

Consistently, HSP27 silencing diminished expression of key mTORC1 downstream effectors, including 4E-BP1/p-4E-BP1 and S6K1/p-S6K1 (Fig. 2D, Supplementary Material 2- Figure S2B). Similar effects were observed with J2, a small-molecule HSP27 inhibitor that impairs its chaperone function by promoting abnormal dimer formation. J2 treatment reduced levels of RAPTOR, 4E-BP1, and S6K1 (Fig. 2E, Supplementary Material 2-Figure S2C). Importantly, these regulatory effects occurred at the protein level without corresponding changes in mRNA expression (Fig. 2F), indicating that HSP27 promotes mTOCR1 activation through stabilization of these three proteins.

### HSP27 stabilizes RAPTOR by preventing proteasomal degradation

**Fig. 3:**
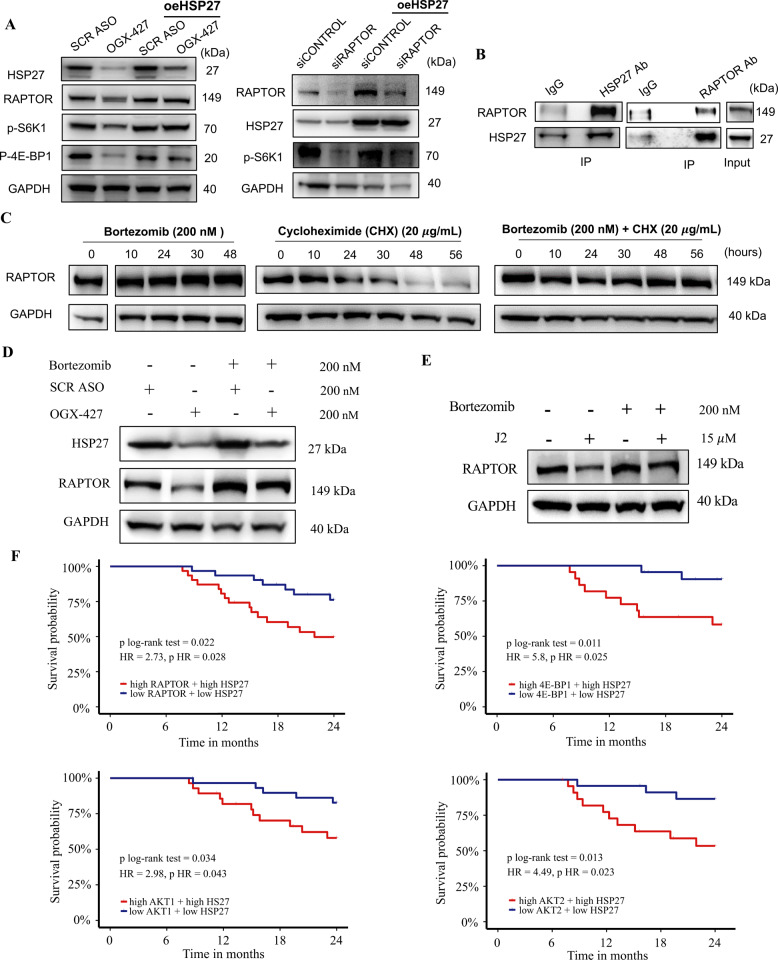
HSP27 stabilizes RAPTOR by preventing its proteasomal degradation in CRPC. **A** HSP27 regulates mTORC1 signaling through RAPTOR in PC-3 cells. HSP27 knockdown using OGX-427 reduced RAPTOR expression and decreased phosphorylation of mTORC1 effectors S6K1 and 4E-BP1 in parental PC-3 cells, whereas HSP27 overexpression restored RAPTOR and downstream signaling (left panel). Reciprocal experiments show that HSP27 re-expression rescued RAPTOR levels and p-S6K1 following RAPTOR silencing (right panel), indicating that HSP27 acts upstream of RAPTOR to control mTORC1 activity. **B** Co-immunoprecipitation (co-IP) assay confirming the physical interaction between endogenous HSP27 and RAPTOR in PC-3 cells. **C** PC-3 cells were treated with cycloheximide (CHX; 20 µg/mL), bortezomib (200 nM), or both, and RAPTOR protein levels were monitored over 10 to 56 h. CHX treatment induced time-dependent RAPTOR degradation, while bortezomib treatment led to RAPTOR accumulation. Co-treatment with CHX and bortezomib prevented RAPTOR degradation, indicating that RAPTOR is primarily turned over by the proteasome. **D-E** HSP27 protects RAPTOR’s stability against proteasome degradation by its chaperone activity. PC-3 cells were treated with HSP27 inhibitors (OGX-427 or J2) alone or in combination with bortezomib. Western blotting showed that bortezomib treatment restored RAPTOR protein levels following HSP27 inhibition, demonstrating that HSP27 protects RAPTOR from proteasomal degradation. **F** Kaplan–Meier survival analysis of overall survival in 101 patients with metastatic castration-resistant prostate cancer (mCRPC) using transcriptomic data from the West Coast Prostate Cancer Dream Team (Stand Up To Cancer–Prostate Cancer Foundation cohort). Hazard ratios (HRs) were calculated using Cox proportional hazards regression models, and differences between survival curves were evaluated by the log-rank test. Patients were stratified based on high versus low co-expression of HSP27 (HSPB1) with key mTORC1 pathway components: RAPTOR**,** 4E-BP1**,** AKT1**,** and AKT2**.** High co-expression of HSP27 with each of these genes was significantly associated with poorer survival within the first 24 months (log-rank *p*-values: 0.022, 0.011, 0.034, and 0.013, respectively). Results with *P* < 0.05 were considered statistically significant

Given RAPTOR's key role as a scaffolding subunit of mTORC1 and its identification as an HSP27 interactor in CRPC cells, we further studied the functional relationship between HSP27 and RAPTOR. OGX-427–mediated HSP27 knockdown reduced RAPTOR expression and phosphorylation of S6K1 and 4E-BP1, whereas HSP27 overexpression restored RAPTOR and downstream signaling. Reciprocal experiments showed that HSP27 re-expression rescued RAPTOR levels and p-S6K1 following RAPTOR silencing, confirming that HSP27 acts upstream of RAPTOR. These reciprocal rescue experiments establish a direct functional link between HSP27 and RAPTOR, demonstrating that HSP27 controls mTORC1 activity rather than merely correlating with pathway activation (Fig. 3A).

The interaction between HSP27 and RAPTOR was confirmed by co-immunoprecipitation (co-IP) (Fig. 3B, Supplementary Material 2- Figure S2D). To determine whether RAPTOR is regulated via proteasomal degradation, PC-3 cells were treated with cycloheximide (CHX; protein synthesis inhibitor), bortezomib (proteasome inhibitor), or both, and RAPTOR levels were monitored over 10 to 56 hours. CHX treatment led to a time-dependent decrease in RAPTOR, while bortezomib treatment caused its accumulation. Importantly, co-treatment with CHX and bortezomib preserved RAPTOR levels (Fig. 3C, Supplementary Material 2 Figure S2E), indicating that RAPTOR is primarily degraded through the proteasome pathway.

We next investigated whether HSP27 protects RAPTOR from proteasomal degradation. Western blot analysis revealed that treatment with bortezomib following HSP27 inhibition either by OGX-427 or J2 restored RAPTOR protein levels (Fig. 3D&E, Supplementary Material 2 Figure S2 F). Together, these findings demonstrate that HSP27 binds and stabilizes RAPTOR, shielding it from proteasomal degradation and thereby sustaining mTORC1 signaling in CRPC cells.

### Clinical association between HSP27 and mTORC1 signalling in metastatic CRPC patients

To substantiate the clinical relevance of the HSP27-mTORC1 axis, we performed a Kaplan–Meier survival analysis using mRNA expression data from 101 metastatic castration-resistant prostate cancer patients, derived from the West Coast Prostate Cancer Dream Team project, Stand Up 2 Cancer and the Prostate Cancer Foundation [21]. Patients with simultaneous high expression of HSP27 and key mTORC1-associated genes such as RAPTOR (HR = 2.73, log-rank *p* = 0.022), 4E-BP1 (HR = 5.8, log-rank *p* = 0.011), AKT1 (HR = 2.98, log-rank *p* = 0.034), or AKT2 (HR = 4.49, log-rank *p* = 0.013) exhibited significantly reduced survival within the first 24 months compared to patients with low co-expression of these gene pairs (Fig. 3F). Moreover, patients with concurrent high HSP27 and low PTEN expression also showed a trend toward worse outcomes (log-rank test *p* = 0.058). In contrast, no significant survival difference was observed in patients co-expressing high levels of HSP27 and either RICTOR or MTOR (Supplementary Material 2 Figure S3).

These findings highlight a clinically meaningful association between elevated HSP27 expression and key components of the mTORC1 signalling pathway, supporting its role in disease progression and poor prognosis in metastatic CRPC.

### Synergistic antitumor effects of OGX-427 in combination with mTOR inhibitors in PC-3 CRPC cells

Given that HSP27 and mTOR inhibitors (e.g., Everolimus, Sapanisertib) modulate mTOR signaling via distinct mechanisms, we hypothesized that co-targeting HSP27 and mTOR could enhance therapeutic efficacy in CRPC. To test this, PC-3 cells were treated with OGX-427 in combination with either Sapanisertib or Everolimus at a fixed 2:1 ratio, and drug interactions were evaluated using the Chou–Talalay method via CalcuSyn software (see Methods).

**Fig. 4:**
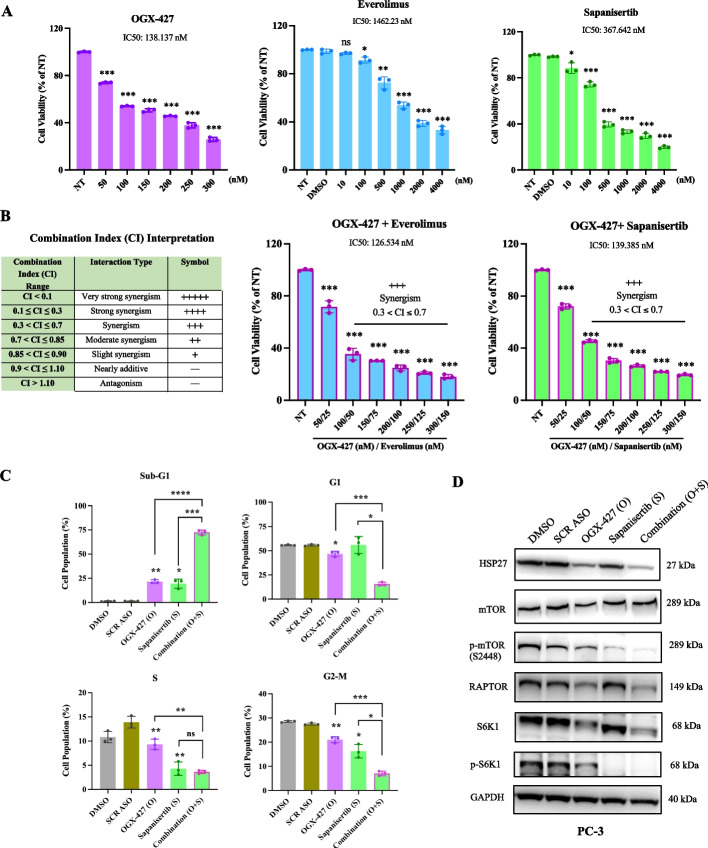
Synergistic antitumor effects of OGX-427 combined with mTOR inhibitors in CRPC PC-3 cells. **A** In PC-3 cells, OGX-427, Everolimus and Sepanisertib inhibited cell proliferation in a dose-dependent manner with IC₅₀ values of 138.14 nM (OGX-427), 1462.23 nM (Everolimus), and 367.64 nM (Sapanisertib), respectively. **B** Combination treatments at a fixed 2:1 ratio of OGX-427 with Everolimus or Sapanisertib reduced the IC₅₀ values and resulted in combination index (CI) values ranging from 0.3 to 0.7, indicating synergistic inhibition of cell proliferation. **C** Cell cycle analysis by flow cytometry shows that co-treatment with OGX-427 and Sapanisertib markedly increased the sub-G1 apoptotic population (72.44%) compared to OGX-427 (21.57%) or Sapanisertib (19.51%) alone, while reducing the proportions of cells in G1, S, and G2/M phases. **D** Western blot analysis shows enhanced inhibition of mTORC1 signaling by combination treatment, with reduced levels of phosphorylated mTOR (Ser2448), RAPTOR, and total and phosphorylated S6K1 compared to monotherapies.The mean values (*n* = 3) with SEM (Standard Error of Mean) are shown, and the independent sample t-test was carried out to compare the means between two groups. Results with *P* < 0.05 were considered statistically significant and are indicated by *, *P* < 0.05; **, *P* < 0.01; ***, *P* < 0.001, and ****, *P* < 0.0001, ns: non-significant

As shown in Fig. 4A, the individual IC₅₀ values were 138.1 nM for OGX-427, 1462.2 nM for Everolimus, and 367.6 nM for Sapanisertib. Co-treatment with OGX-427 reduced the IC₅₀ values of the combinations to 126.5 nM with Everolimus and 139.4 nM with Sapanisertib (Fig. 4B). Notably, the calculated combination index (CI) values were within 0.3 and 0.7 (synergism), indicating synergy between OGX-427 and mTOR inhibitors in suppressing tumor cell proliferation (Fig. 4B). This combinatorial effect resulted in significant dose reductions. To achieve 90% inhibition of PC-3 cell viability, 1185.9 nM of OGX-427 or 15,276.3 nM of Sapanisertib were required as monotherapies. In contrast, the same effect was obtained with only 493.1 nM of OGX-427 plus 246.6 nM of Sapanisertib when combined—a 58.4% and 98.4% dose reduction, respectively (Table 1). These results suggest that combined inhibition of HSP27 and mTORC1 represents a potent strategy to enhance anti-tumor responses while minimizing drug exposure.

**Table 1:** Synergistic anti-tumor effects of the combinational drugs (OGX-427 and mTORi) in PC-3 cells

Fa (Percentage of death cells)	Monotherapy			Combination (O + S)		Combination (O + E)	
	**OGX-427 (O)** **(nM)**	**Sapanisertib (S)** **(nM)**	**Everolimus (E)** **(nM)**	**OGX-427** **(nM)**	**Sapanisertib** **(nM)**	**OGX-427** **(nM)**	**Everolimus** **(nM)**
50%	138.14	367.64	1462.23	92.92 (- 32.7%)	46.46 (−87.4%)	84.36 (−39%)	42.18 (−97%)
75%	404.74	2369.85	6335.96	214.06 (−47.1%)	107.03 (−95.5%)	197.87 (−51%)	98.93 (−98%)
90%	1185.88	15,276.3	27,454.2	493.11 (−58.4%)	246.55 (−98.4%)	464.12 (−61%)	232.06 (−99.1%)
95%	2463.63	54,256.9	74,425.7	869.8 (−64.7%)	434.92 (−99.2%)	828.83 (−66%)	414.41 (−99.5%)

Cell cycle analysis revealed that co-treatment with OGX-427 and Sapanisertib significantly increased the sub-G1 population, a hallmark of apoptotic cell death, while reducing the proportion of cells in the G1, S, and G2/M phases. These results indicate that the combination induces stronger cell cycle disruption and promotes apoptosis more effectively than either agent alone (Fig. 4C). Consistently, western blot analysis demonstrated enhanced inhibition of HSP27 and mTORC1 signaling with the combination treatment. Co-administration of OGX-427 and Sapanisertib led to a more pronounced reduction in the expression of phosphorylated mTOR (Ser2448), RAPTOR, total and phosphorylated S6K1 compared to monotherapies (Fig. 4D). Together, these findings support the synergistic effects of dual HSP27 and mTOR inhibition in disrupting tumor cell survival mechanisms in CRPC.

### Dual inhibition of HSP27 and mTOR signaling synergistically suppresses growth in Patient-Derived Organoids (PDOs)

**Fig. 5:**
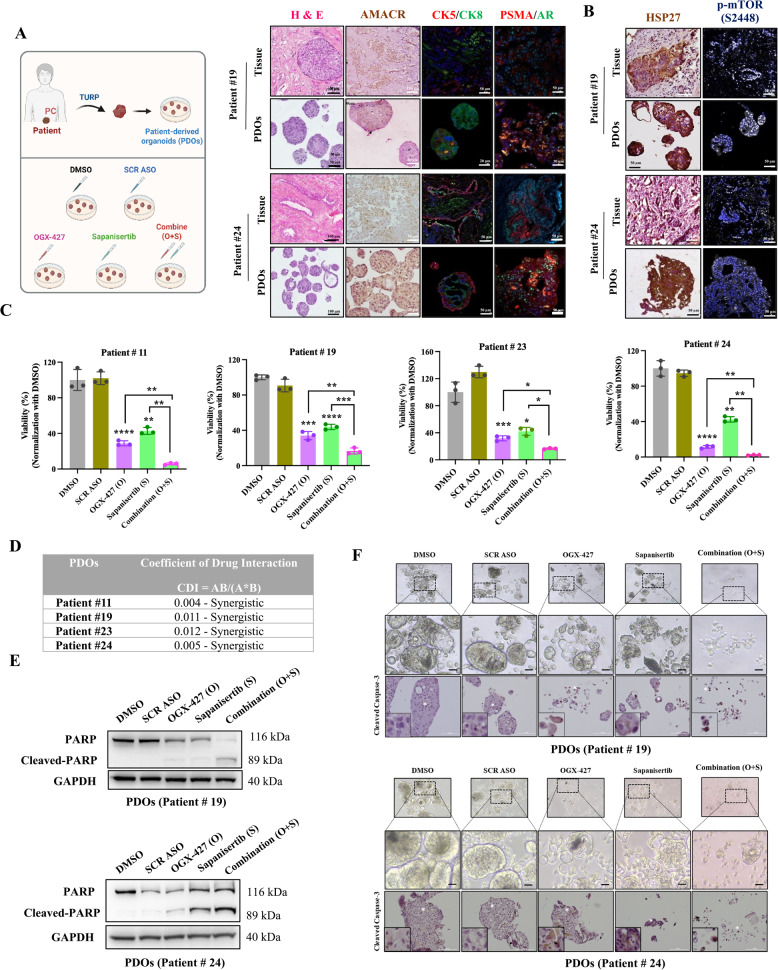
Combination of OGX-427 and Sapanisertib shows a synergistic anti-tumor effect in advanced PC patient-derived organoids (PDOs) model. **A** Representative Hematoxylin and Eosin (H&E) staining of PDOs and matched tumor tissues demonstrating preserved histopathological architecture. Immunofluorescence analysis shows co-expression of basal (CK5) and luminal (CK8) epithelial markers, along with prostate cancer-associated markers PSMA, AR, and AMACR, confirming molecular fidelity of PDOs to original tumors. **B** PDOs exhibit high expression of HSP27 and phosphorylated mTOR (Ser2448), indicating active HSP27/mTOR signaling. **C-D** The organoids were cultured in 96-well plates and treated with OGX-427 (250 nM), Sapanisertib (125 nM), or a combination of both compounds. After three days of treatment, the cell viability assay was performed. Combination treatments yielded Coefficients of Drug Interaction (CDI) significantly less than 1 (0.004, 0.011, 0.012, and 0.005), indicating strong synergistic inhibition of organoid viability. CDI value < 1 indicates synergy, = 1 indicates additive effects, and > 1 indicates antagonism. **E** Western blot analysis demonstrates that combination therapy more strongly induce apoptosis by activating PARP (Cleaved-PARP) compare to vehicle controls or monotherapy. **F** The representative IHC images of the cleaved caspase-3 staining were shown with the scale bar of 100 μm, while the representative organoid images taken under microscopy were shown with scale bar of 25 μm. The combination of these two drugs enhanced the apoptosis (cleaved caspase-3) compared to the monotherapies on the PDOs model. The mean values (*n* = 3) with SEM (Standard Error of Mean) are shown, and the independent sample t-test was carried out to compare the means between two groups. Results with *P* < 0.05 were considered statistically significant and are indicated by *, *P* < 0.05; **, *P* < 0.01; ***, *P* < 0.001, and ****, *P* < 0.0001, ns: non-significant

In our laboratory, we have developed patient-derived organoids (PDOs) from metastatic prostate cancer (PC) tissues (Tran T.T. et al., submitted manuscript). To assess how faithfully these PDOs recapitulate the original tumor characteristics, we analyzed their morphological and molecular features. Hematoxylin and Eosin (H&E) staining revealed that the organoids retained key histopathological structures of the corresponding tumor tissues, as confirmed by a pathologist. Immunofluorescence analysis demonstrated the co-expression of basal (CK5) and luminal (CK8) epithelial markers, indicating preservation of prostate epithelial lineage. Furthermore, PDOs maintained the expression of critical PC-associated markers, including Prostate-Specific Membrane Antigen (PSMA), Androgen Receptor (AR), and Alpha-Methylacyl-CoA Racemase (AMACR), further validating their molecular similarity to the original tumors (Fig. 5A). Organoids derived from these patients also exhibited high levels of HSP27 and phosphorylated mTOR at Ser2448 (Fig. 5B).

To evaluate the therapeutic potential of targeting the HSP27/mTOR axis, PDOs were cultured in low-adherence 96-well plates and treated with OGX-427 (250 nM), Sapanisertib (125 nM), or their combination. Drug interaction was assessed using the Coefficient of Drug Interaction (CDI) [30], calculated from cell viability assays in PDOs derived from four patients. A CDI value < 1 indicates synergy, = 1 indicates additive effects, and > 1 indicates antagonism. All four PDO samples showed CDI values well below 1 (0.004, 0.011, 0.012, and 0.005), indicating a strong synergistic anti-tumor effect of the combined treatment (Fig. 5C–D). Furthermore, combination therapy resulted in increased apoptosis, as evidenced by elevated PARP cleavage in Western blot analysis and enhanced immunostaining for cleaved Caspase-3 (Fig. 5E–F). Collectively, these findings support that dual inhibition of HSP27 and mTOR elicits a synergistic therapeutic effect in PDOs derived from metastatic PC, highlighting a promising strategy for overcoming treatment resistance in this setting.

### Enhanced anti-tumor effects of OGX-427 and Sapanisertib combination in vivo using PC-3 xenograft model

**Fig. 6:**
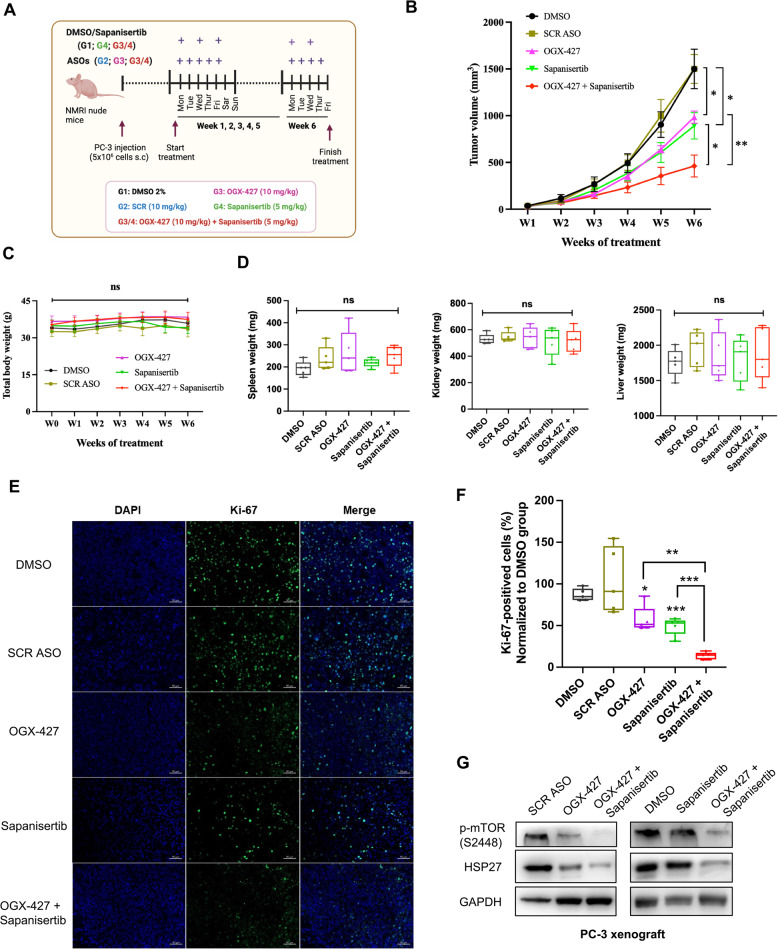
Combination of OGX-427 and Sapanisertib shows a stronger inhibition of tumor growth with no body toxicity observed. **A** Schematic picture illustrates the treatment groups: DMSO 2% (G1), SCR ASO (G2), OGX-427 (G3), Sapanisertib (G4), combination of OGX-427 and Sapanisertib (G3/4), and schedule of injection for mice in different groups for 6 weeks. **B** Mean tumor volume growth curve of PC-3 xenografted tumors. The 6-week-old male NMRI nude mice harboring PC-3-derived xenografted tumor (5–6 mice/group) were intraperitoneally injected three times per week (Monday, Wednesday, Friday) with DMSO 2%, 5 mg/kg Sapanisertib, or were intraperitoneally injected daily (from Monday to Friday) with 10 mg/kg SCR ASO or OGX-427 for 6 weeks. Mice in the combination group were injected with both Sapanisertib and OGX-427 similar to mice injected with only Sapanisertib or OGX-427. Tumor volume (mm^3^) was measured weekly using a calliper in three perpendicular dimensions (X = width, Y = length, Z = depth) and calculated following the formula X*Y*Z*0.5236. Mice treated with the combination of OGX-427 and Sapanisertib show a higher tumor growth inhibition compared with the group of Vehicle controls or monotherapy of OGX-427 or Sapanisertib. **C-D** Evaluation of body’s toxicity by measuring total body weight following 6 weeks of treatment and weight of liver, kidney, and spleen at the end of the experiment. Data (Mean ± SEM, *n* = 5) shows no body toxicity observed when combined OGX-427 and Sapanisertib, compared to control groups or OGX-427 or Sapanisertib group. **E** Representative pictures of Ki-67 (a proliferation marker) immunofluorescence staining in formalin-fixed tumor xenograft harvested from mice in 5 groups of treatment at the end of the experiment. **F** Quantification of Ki-67-positive cells (Mean ± SEM) shows a low percentage of proliferation cells in tumor xenograft of mice treated with the combination of OGX-427 and Sapanisertib, compared to control groups or OGX-427 or Sapanisertib group, (*n* = 5). **G** Representative Western blot shows expression of HSP27 and p-mTOR (S2448) in tumor xenograft of mice treated with combination therapy decreased, compared to mice treated with either OGX-427 or Sapanisertib. The mean values with SEM (Standard Error of Mean) are shown, and the independent sample t-test was carried out to compare the means between two groups. Results with *P* < 0.05 were considered statistically significant and are indicated by *, *P* < 0.05; **, *P* < 0.01; ***, *P* < 0.001, and ****, *P* < 0.0001, ns: non-significant

To evaluate the therapeutic potential of dual targeting HSP27 and mTOR in vivo, we tested the combination of OGX-427 and Sapanisertib in a subcutaneous PC-3 xenograft model using immunodeficient mice. Animals were randomized into five treatment groups: (G1) vehicle control (2% DMSO, 100 µL/injection), (G2) scrambled ASO (10 mg/kg), (G3) OGX-427 (10 mg/kg), (G4) Sapanisertib (5 mg/kg), and (G3/4) OGX-427 (10 mg/kg) plus Sapanisertib (5 mg/kg) (Fig. 6A). After six weeks of treatment, the combination of OGX-427 and Sapanisertib resulted in a greater reduction in tumor growth compared to the individual therapies (Fig. 6B), without evidence of systemic toxicity (Fig. 6C&D, Supplementary Material 2- Figure S4&5).

Immunofluorescence analysis of tumor sections revealed a marked decrease in Ki67-positive proliferating cells in the combination group relative to the monotherapy groups, indicating enhanced anti-proliferative effects (Fig. 6E–F). Furthermore, Western blot analysis of tumor lysates showed a more pronounced reduction in phosphorylated mTOR (Ser2448) and HSP27 expression in the combination-treated tumors, confirming effective target engagement and pathway inhibition (Fig. 6G).

## Discussion

Although HSP27 has long been recognized for its role in promoting tumor progression and therapy resistance in prostate cancer, the molecular mechanisms underlying its oncogenic functions remain incompletely understood. In this study, we provide the first comprehensive characterization of the HSP27 interactome in PC and reveal how it evolves along disease progression. Our findings suggest that HSP27 may not only broaden its interaction network in aggressive, CRPC models, but also play a critical role in maintaining cellular homeostasis, metabolic plasticity, and survival under stress (Graphical abstract).

Importantly, we uncover a novel role for HSP27 as a positive regulator of the mTOR signaling pathway, a central node of growth, metabolism, and therapy resistance in advanced cancers [31]. As PC progresses from CSPC to CRPC phenotype, tumor cells become more aggressive by activating adaptive responses and increasingly relying on alternative pathways, notably PI3K/AKT/mTOR signalling [32]. This pathway is frequently altered in CRPC, with pathogenic somatic mutations in related genes detected in 42% of primary tumors and the vast majority of PC metastases [33]. It is also involved in chemoresistance in various cancers, including PC [34].

Our findings expand upon previous work showing that HSP27 regulates 4E-BP1 and eIF4E levels [13], by demonstrating that HSP27 exerts chaperone-mediated stabilization of multiple key mTORC1 components, namely RAPTOR, S6K1, and 4E-BP1 (Graphical abstract). These interactions likely enhance mTORC1 activity and contribute to tumor survival and proliferation in CRPC. These results are consistent with previous reports in colon cancer, where HSP27 knockdown reduced AKT/mTOR phosphorylation and enhanced chemotherapy-induced apoptosis [35]. Crucially, we demonstrate for the first time that co-targeting HSP27 with OGX-427 and mTOR using Sapanisertib or Everolimus yields potent synergistic anti-tumor activity across multiple preclinical models, including CRPC PC-3 cells, PDOs from advanced prostate cancer, and xenograft tumors (Figs. 4, 5 and 6).

Despite mTOR being a well-validated target in oncology, clinical trials of mTOR inhibitors in PC have yielded limited success, largely due to compensatory feedback loops within the PI3K/AKT/mTOR pathway and cross-talk with androgen receptor signaling [32, 36]. Our study suggests that co-targeting HSP27 and mTOR may help overcome these resistance mechanisms via multiple mechanisms:

(i) RAPTOR is essential for the assembly, subcellular localization, and stability of mTORC1, as well as for the recruitment of its downstream substrates [31, 37]. In the present study, we demonstrate that HSP27 stabilizes RAPTOR; therefore, its inhibition may impair mTORC1 assembly and substrate recruitment, thereby potentiating the antitumor effects of mTOR -targeted therapies.
(ii)  As an allosteric inhibitor of mTORC1, first generation of mTOR inhibitors (Rapamycin and its derivatives) exhibits incomplete pathway inhibition [38], as phosphorylation of 4E-BP1 re-emerges after 12 h, restoring cap-dependent translation [39]. HSP27 downregulation suppressed the expression of 4E-BP1 and S6K1 (Fig. 2); therefore, HSP27 inhibition may prevent this rebound, enhance the suppression of protein synthesis when combine with mTORC1 inhibitors.
(iii)  S6K1 phosphorylation by mTORC1 activates multiple negative feedback loops that attenuate PI3K signaling, contributing to resistance to mTOR inhibitors [38]. Our study showed that HSP27 downregulation led to decrease S6K1 expression and phosphorylation. Consequently, inhibition of HSP27 may relieve S6K1-mediated feedback suppression of PI3K signaling, thereby enhancing the anti-tumor efficacy of mTOR inhibitors.

In this study, we identified HSP27 interaction networks and analyzed their biological functions in human prostatic cell lines exhibiting increasing aggressiveness. A limitation of our work is that both CRPC models employed are AR-negative, which may not fully capture AR-dependent mechanisms of resistance. Future investigations applying AP–MS to AR-positive CRPC models such as C4-2 or 22Rv1, as well as to clinical specimens, are warranted to further elucidate HSP27-regulated pathways contributing to androgen signaling independence.

## Conclusion

This study provides new insights into the role of HSP27 in regulating mTORC1 signaling and offers strong preclinical evidence supporting the co-targeting of HSP27 and mTOR as an effective therapeutic strategy for castration-resistant prostate cancer (CRPC). These findings pave the way for future studies to further dissect HSP27-centered protein networks and support clinical evaluation of combination therapies involving OGX-427 and mTOR inhibitors in treatment-resistant prostate cancer.

## Supplementary Information

Supplementary Material 1.

Supplementary Material 2.

Supplementary Material 3.

Supplementary Material 4.

## Data Availability

The mass spectrometry proteomics data have been deposited to the ProteomeXchange Consortium ([www.proteomexchange.org] (http://www.proteomexchange.org)) via the PRIDE partner repository ([https://www.ebi.ac.uk/pride/login] (https://www.ebi.ac.uk/pride/login)) with the dataset identifiers\u0000PXD056858\u0000(Reviewer account details: username: [reviewer_pxd056858@ebi.ac.uk](mailto:reviewer_pxd056858@ebi.ac.uk)/password: IcEyDs5Pjqu0).
