# Supplementary material for: HSP27 functional switch drives castration-resistant prostate cancer via mTOR pathway activation, highlighting promising combination therapies
Source: J Exp Clin Cancer Res. 2026 Mar 25;45:98. doi: 10.1186/s13046-026-03695-6 (PMC13085302; doi:10.1186/s13046-026-03695-6)
Supplement: Supplementary file 2 — Supplementary Material 2. [file 13046_2026_3695_MOESM2_ESM.docx]

**Figure S1.** Bar charts showing GO enrichment analysis of HSP27 interacting-proteins identified in DU-145 (A) and PC-3 (B). Enrichment functional analysis of the HSP27 interaction networks was performed using the ClusterProfiler package in R (p=0,05)

**A**

**B**

**Figure S2. Quantifications of the blots in the Figure 2 and Figure 3**

**mTOR**

**p-mTOR**

**RAPTOR**

**RICTOR**


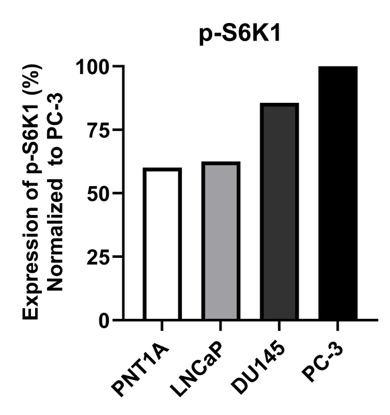

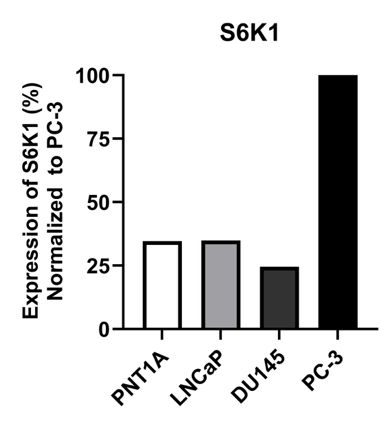

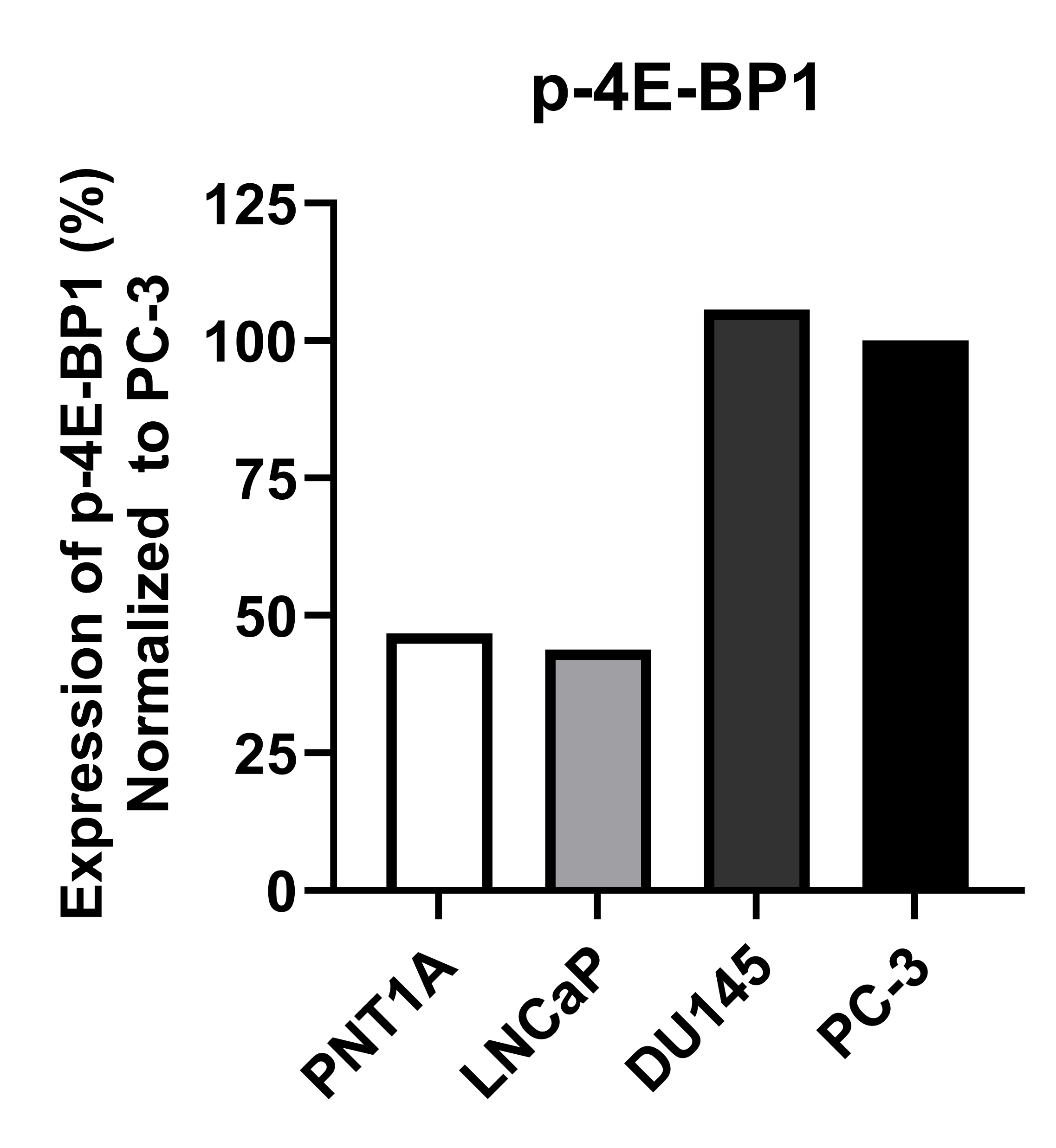

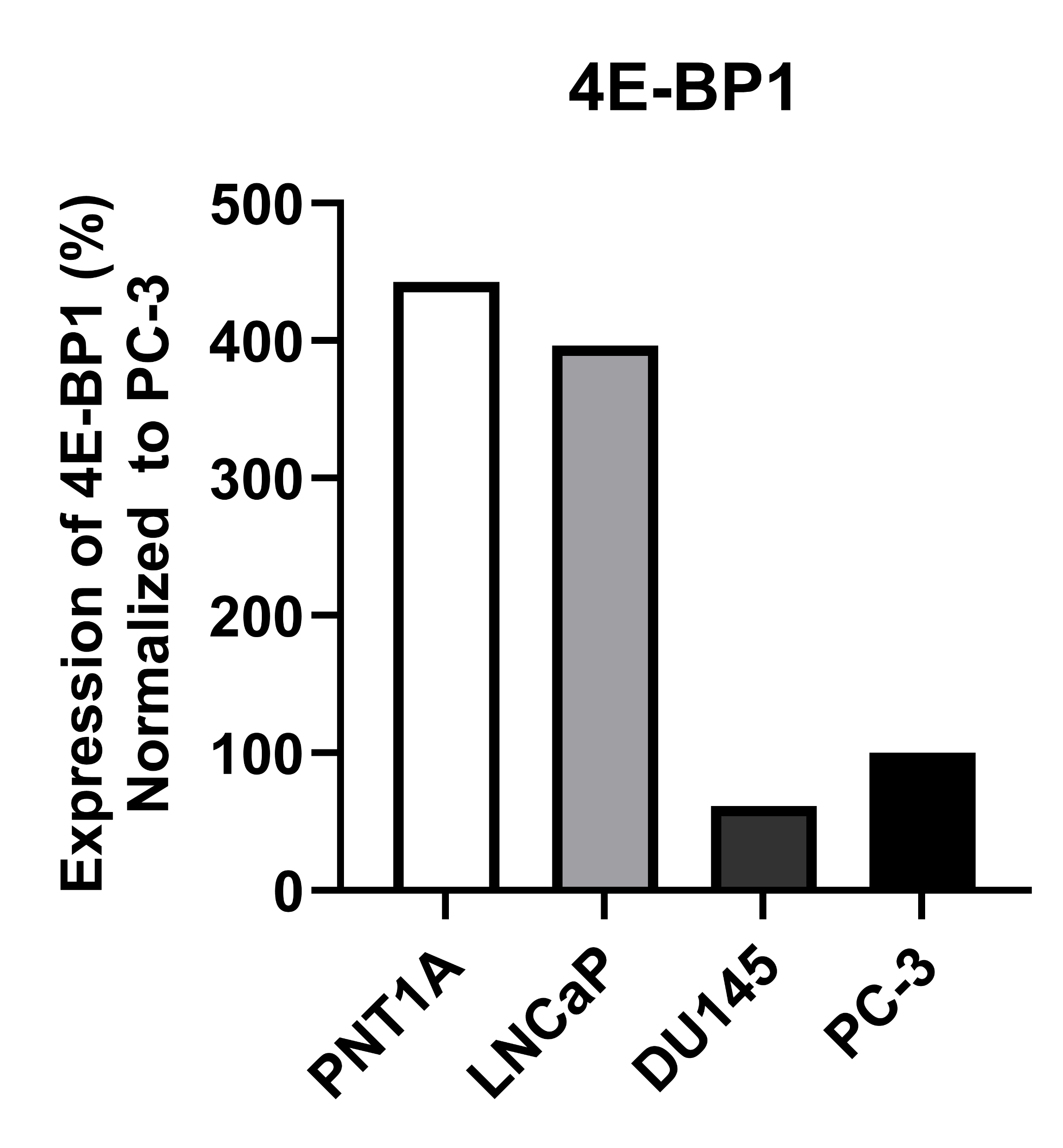


**Relative protein level**

**Relative protein level**

**Relative protein level**

**Relative protein level**

**Relative protein level**

**Relative protein level**

**A.**

**Relative protein level**

**Relative protein level**

**B.**

**S6K1**

**p-S6K1**

**4E-BP1**

**p-4E-BP1**

**Relative protein level**

**Relative protein level**

**C.**

**
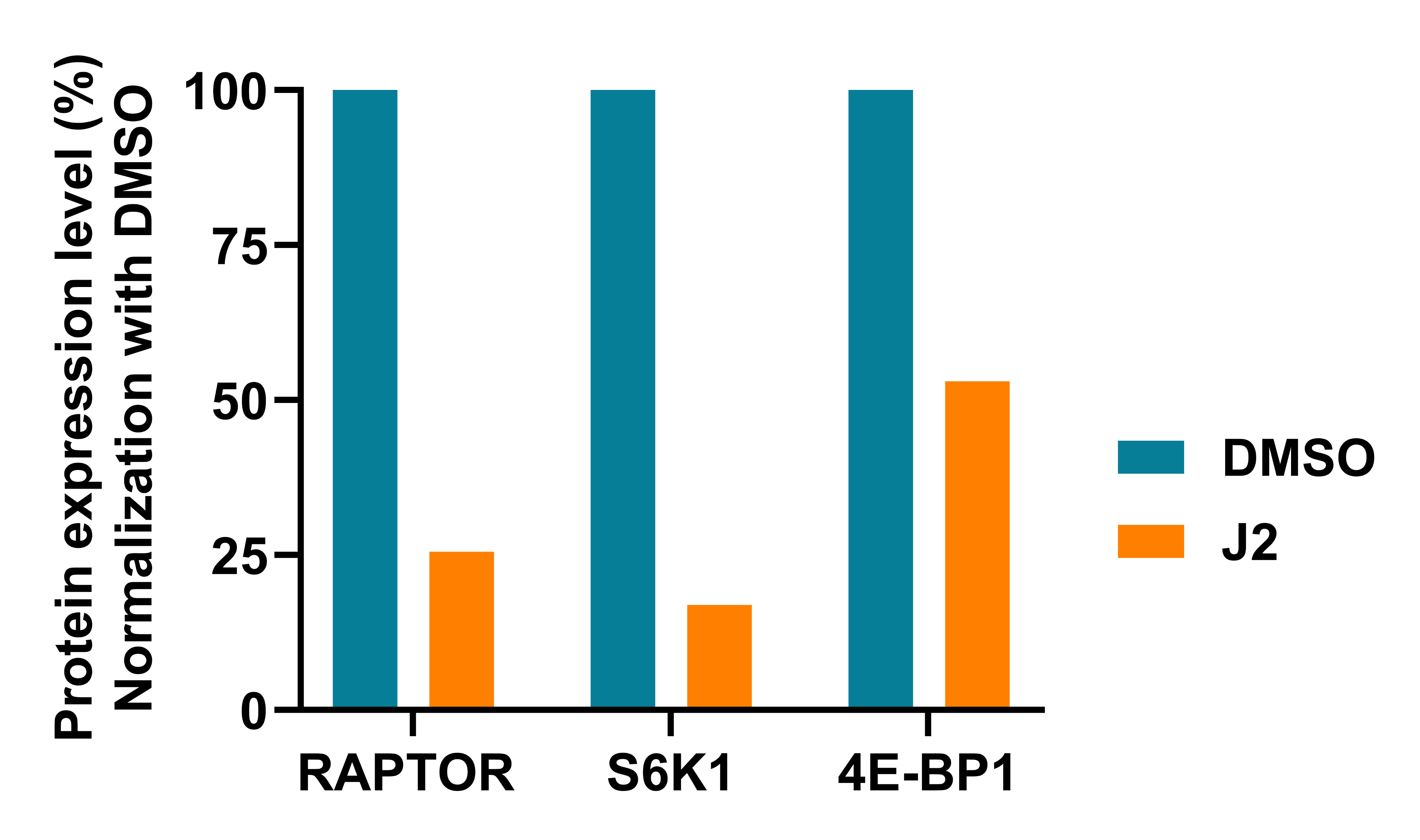
**

**Relative protein level**

**D.**

**
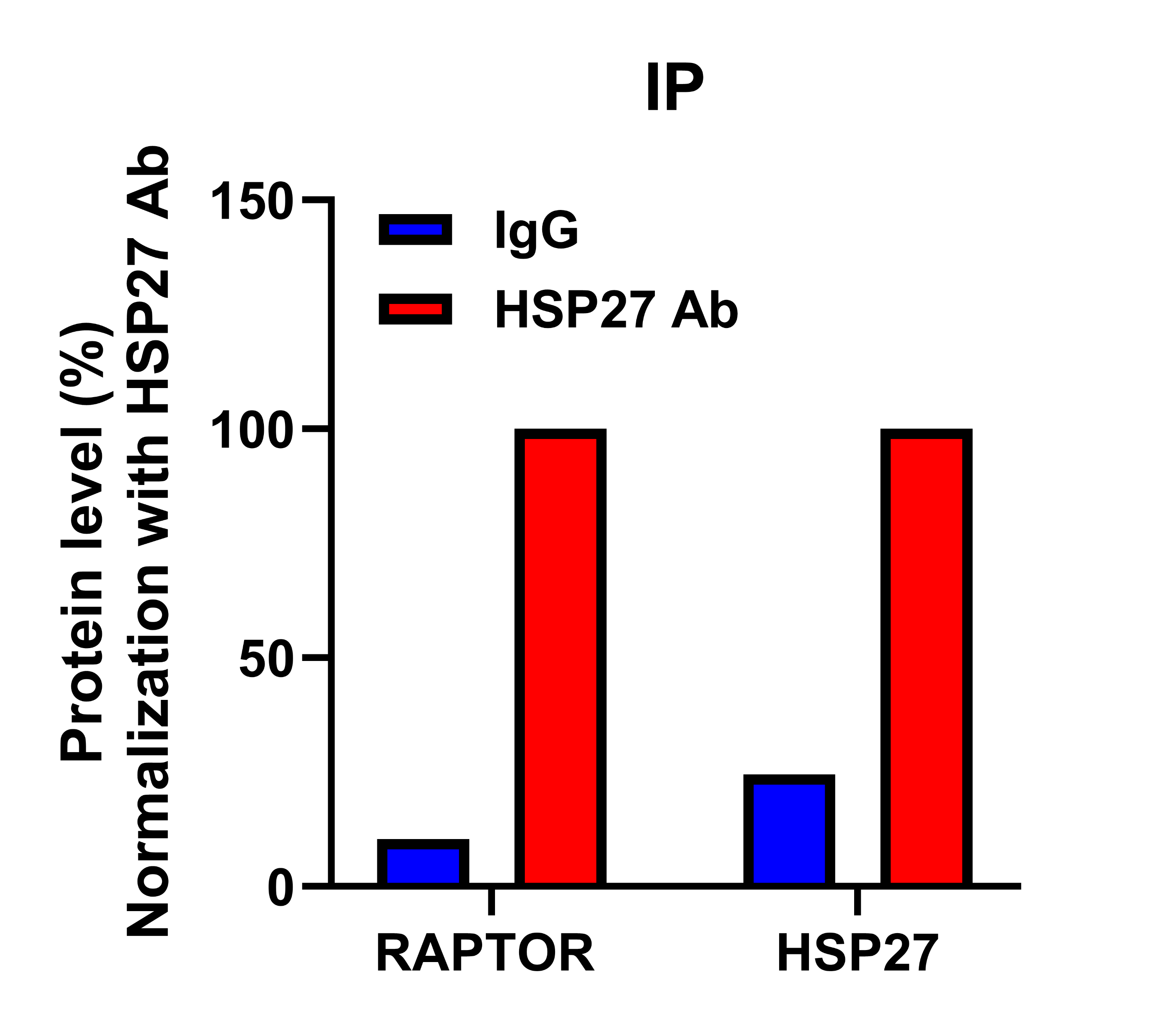
** **
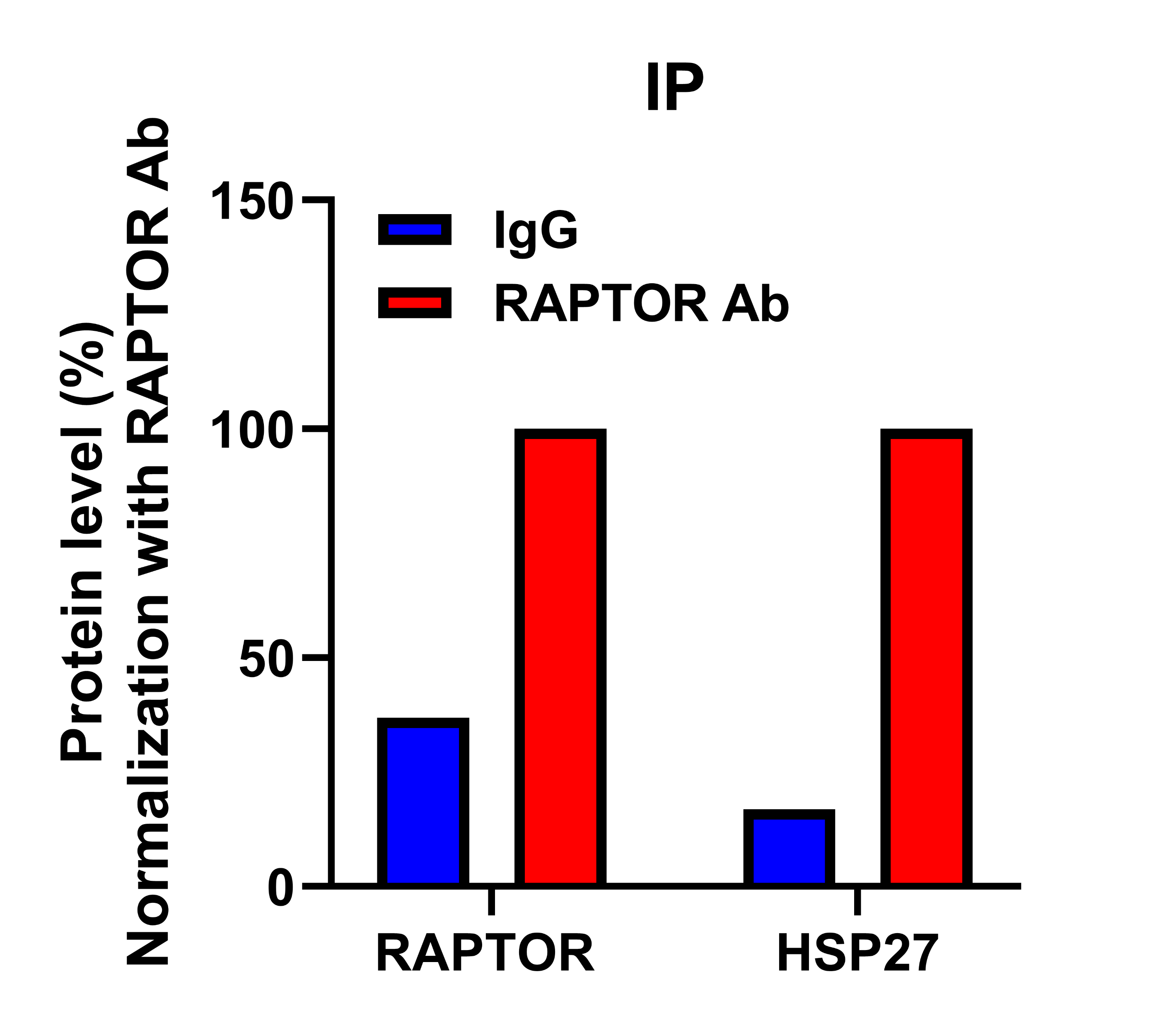
**

**Relative protein level**

**Relative protein level**

**E.**

**
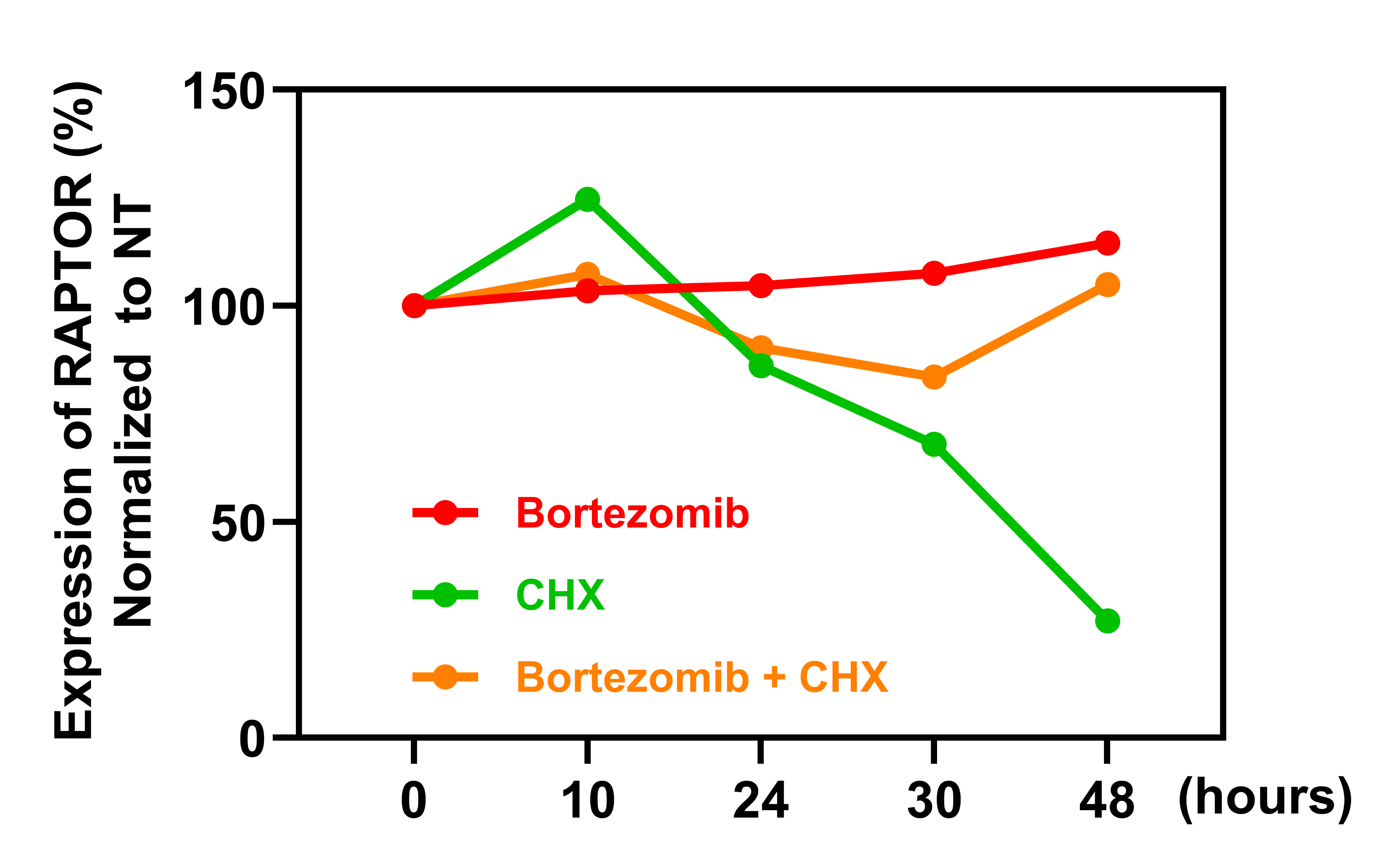
**

**Relative protein level**

**
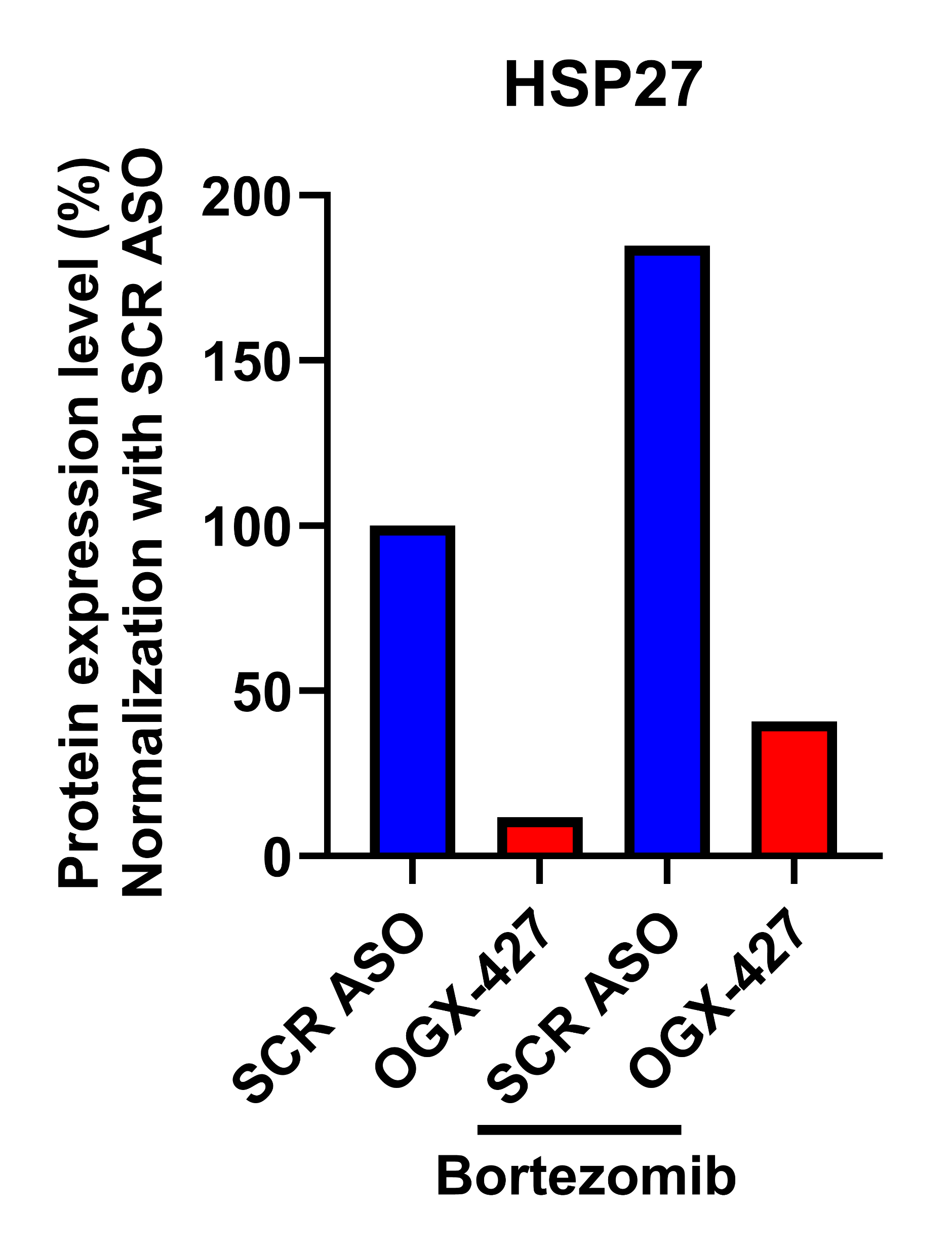
F.**

**
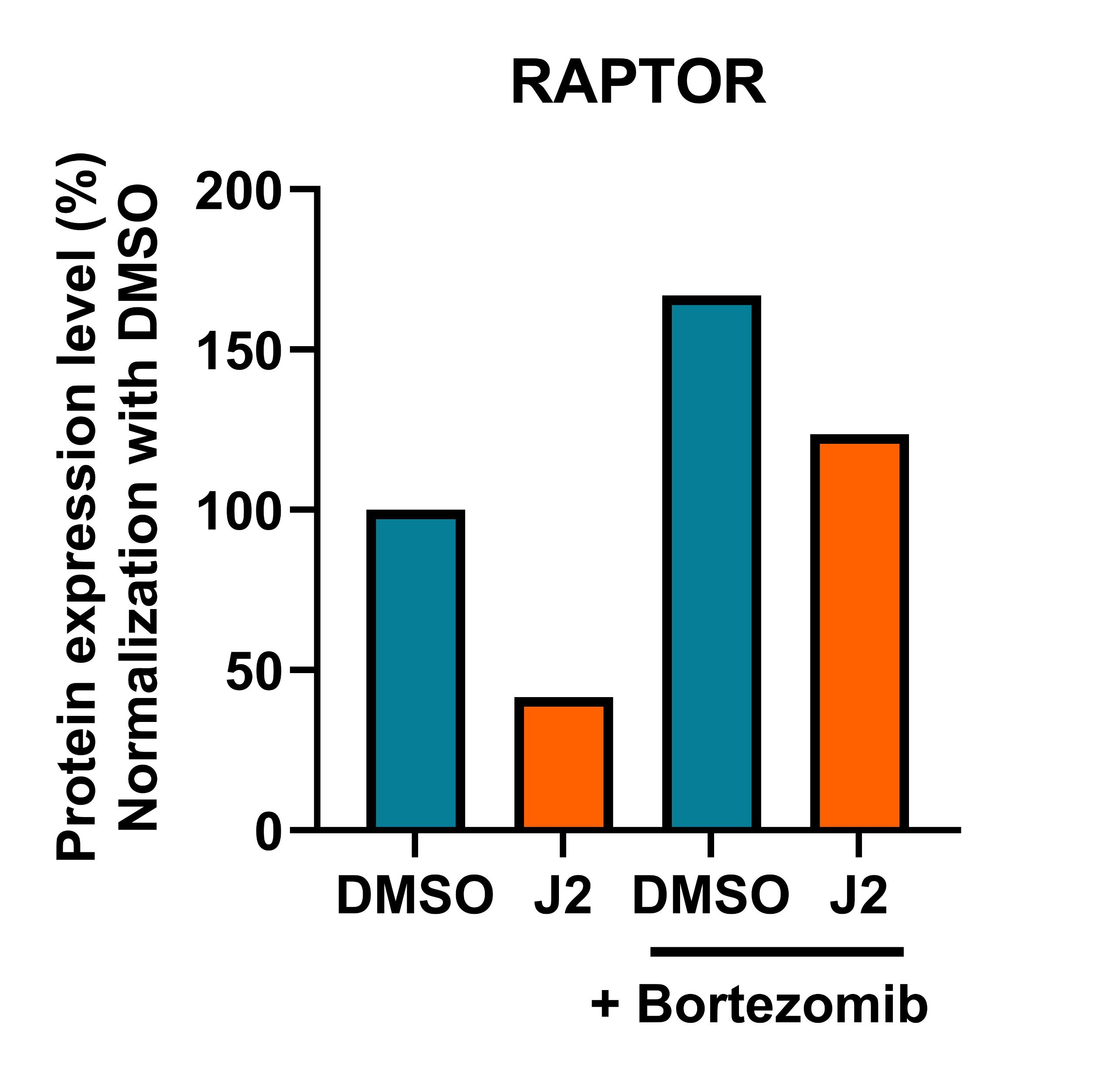

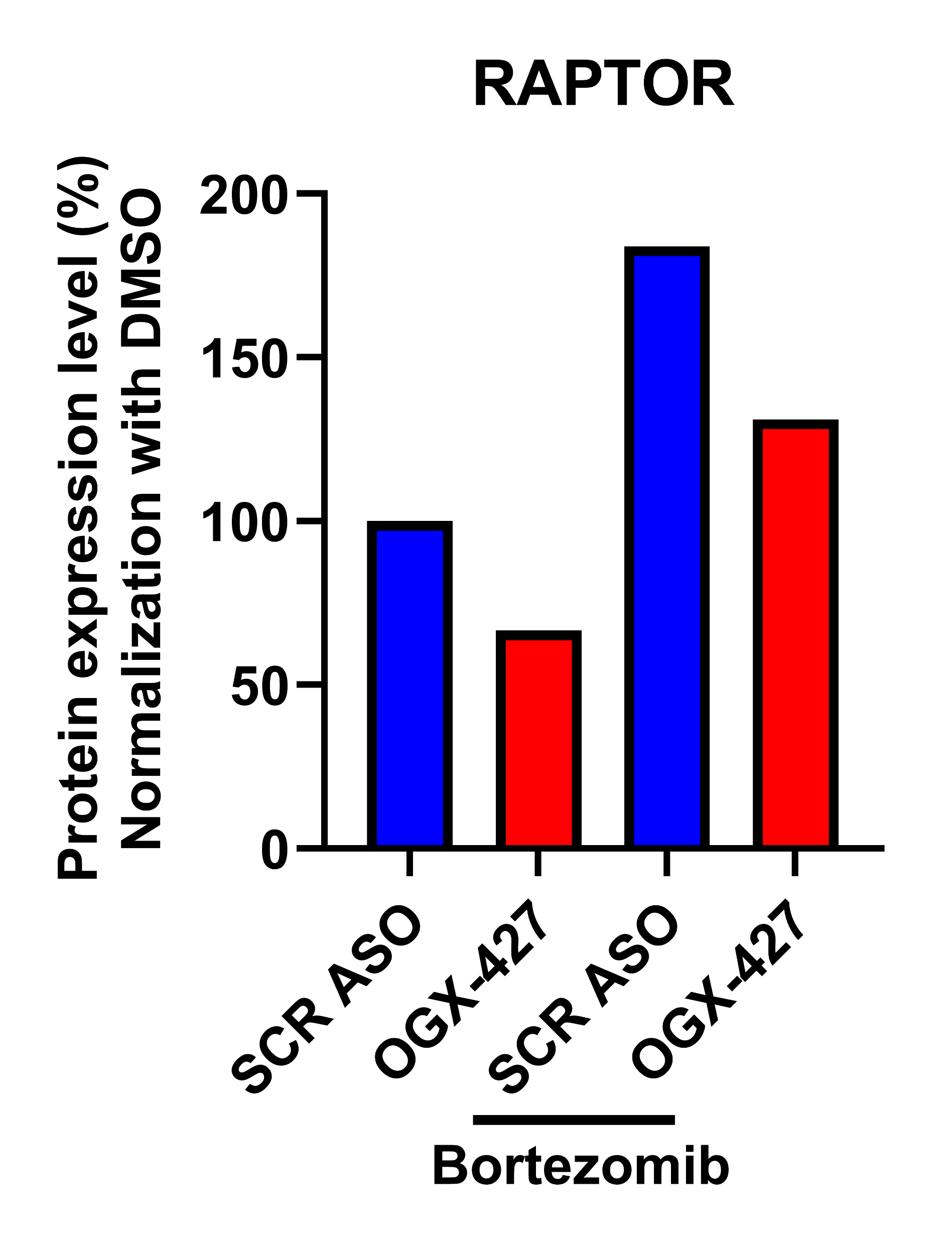
**

**Relative protein level**

**Relative protein level**

**Figure S3. Kaplan-Meier survival analysis in mCRPC patients based on the simultaneous mRNA expression of HSP27 and genes involved in AKT/mTOR pathways.** Data was derived from West Coast Prostate Cancer Dream Team project, Stand Up 2 Cancer and the Prostate Cancer Foundation

**
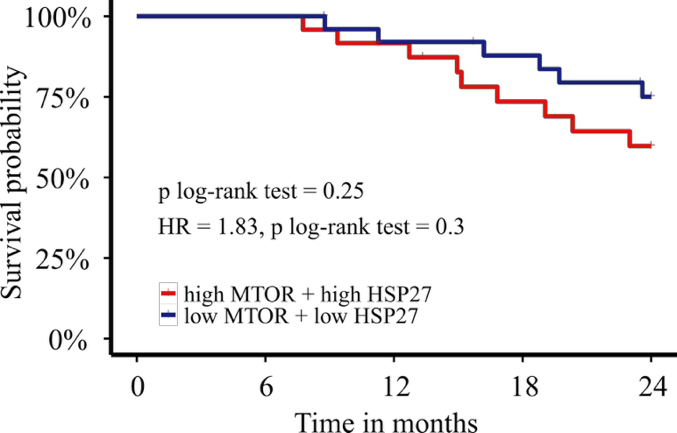

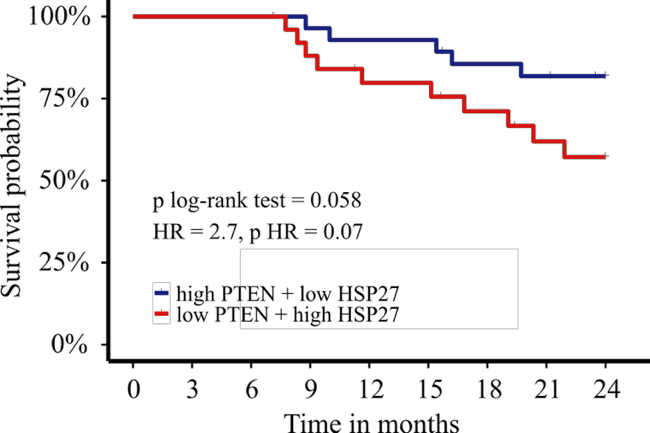
**

**
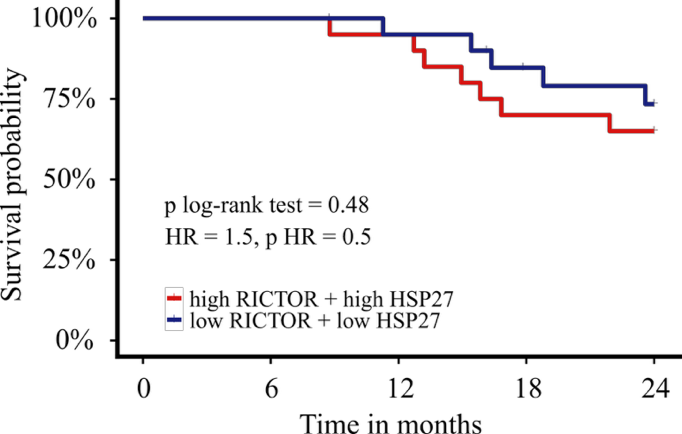
**

**Figure S4. Blood tests assessing systemic toxicity following treatment**. Markers included alanine aminotransferase (ALT) and alkaline phosphatase (ALP) for liver function, as well as blood urea nitrogen (BUN), total protein (TP), and glucose for renal and systemic status. No significant toxicity was observed for the combination treatment compared with monotherapy or control groups.


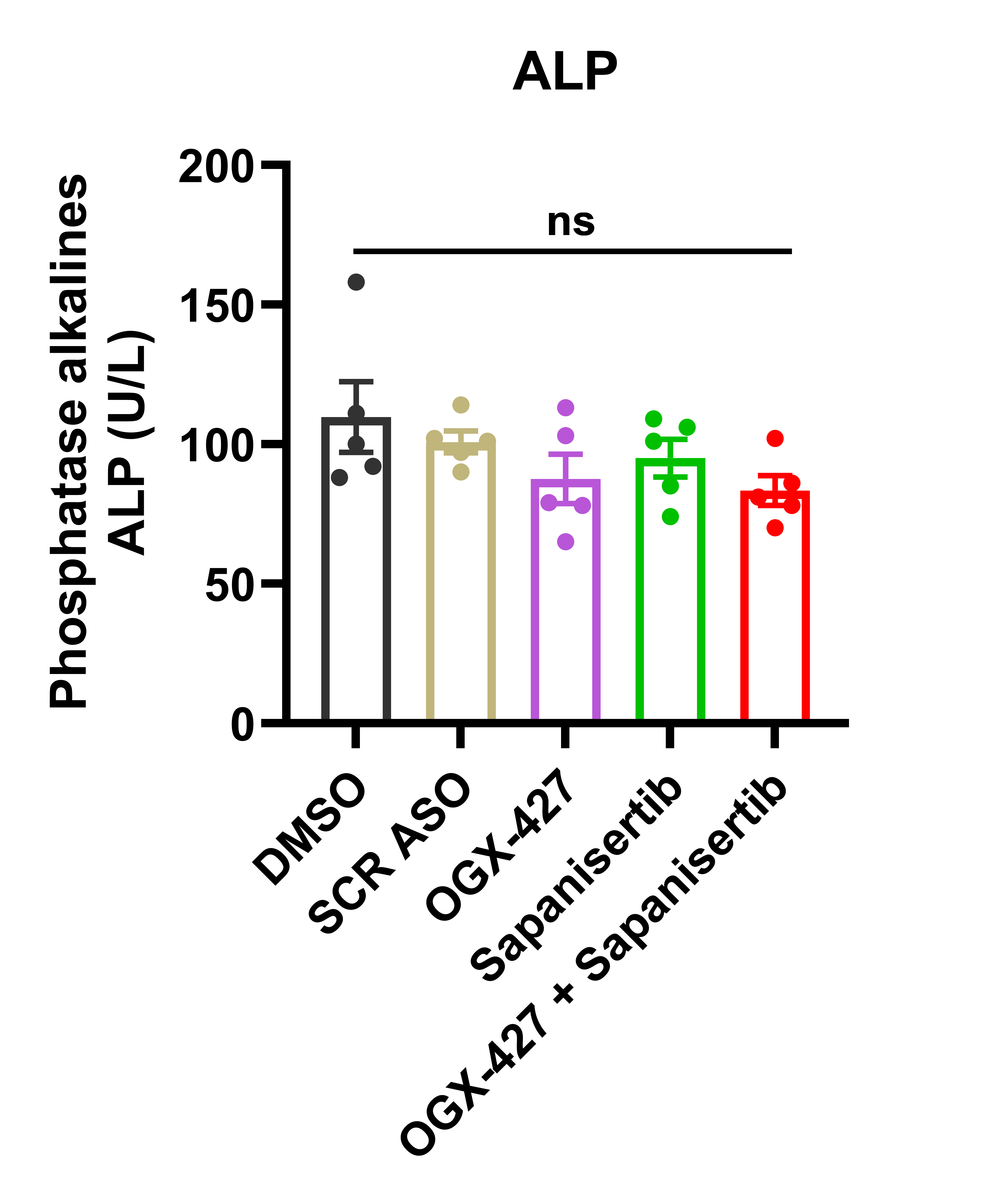

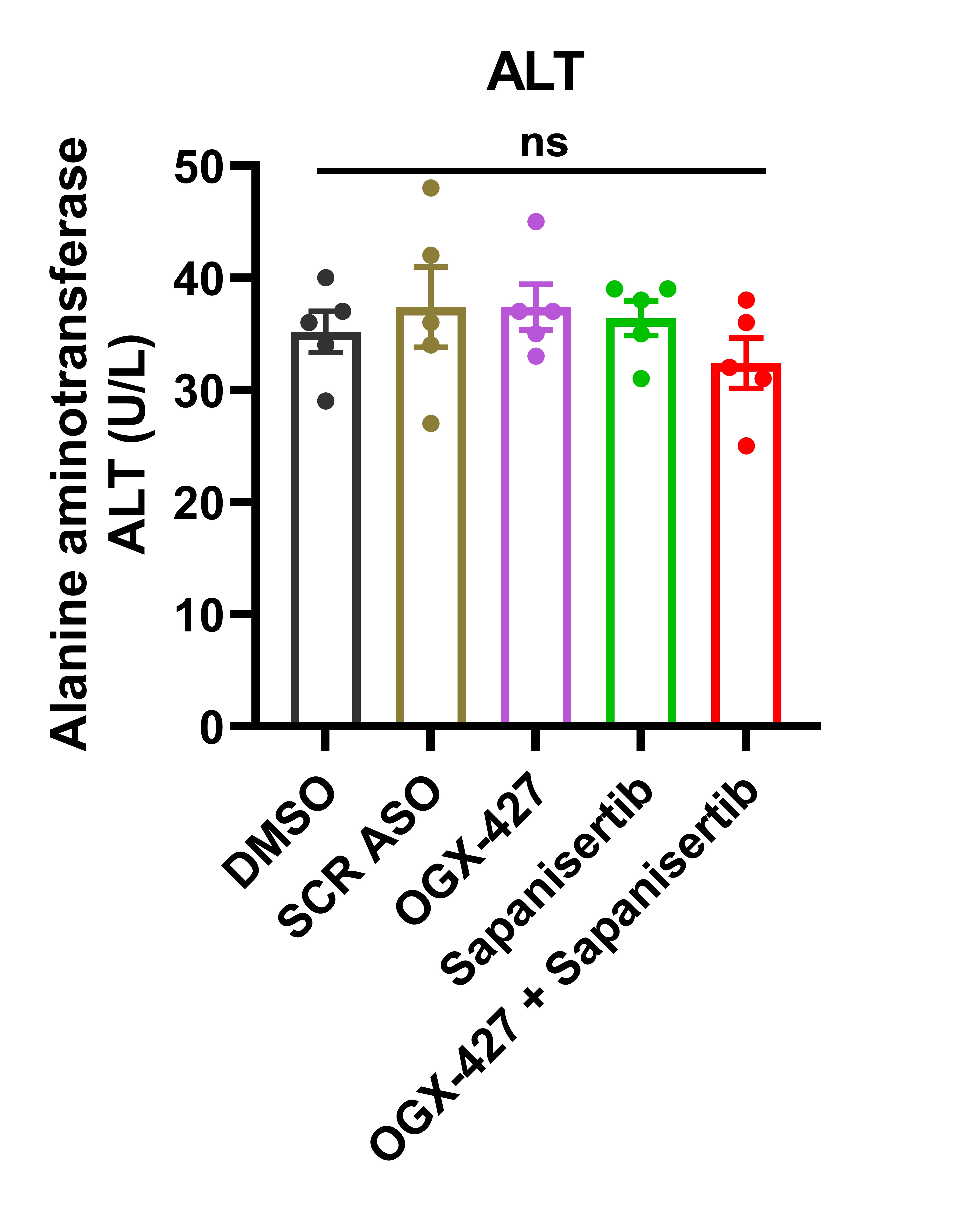

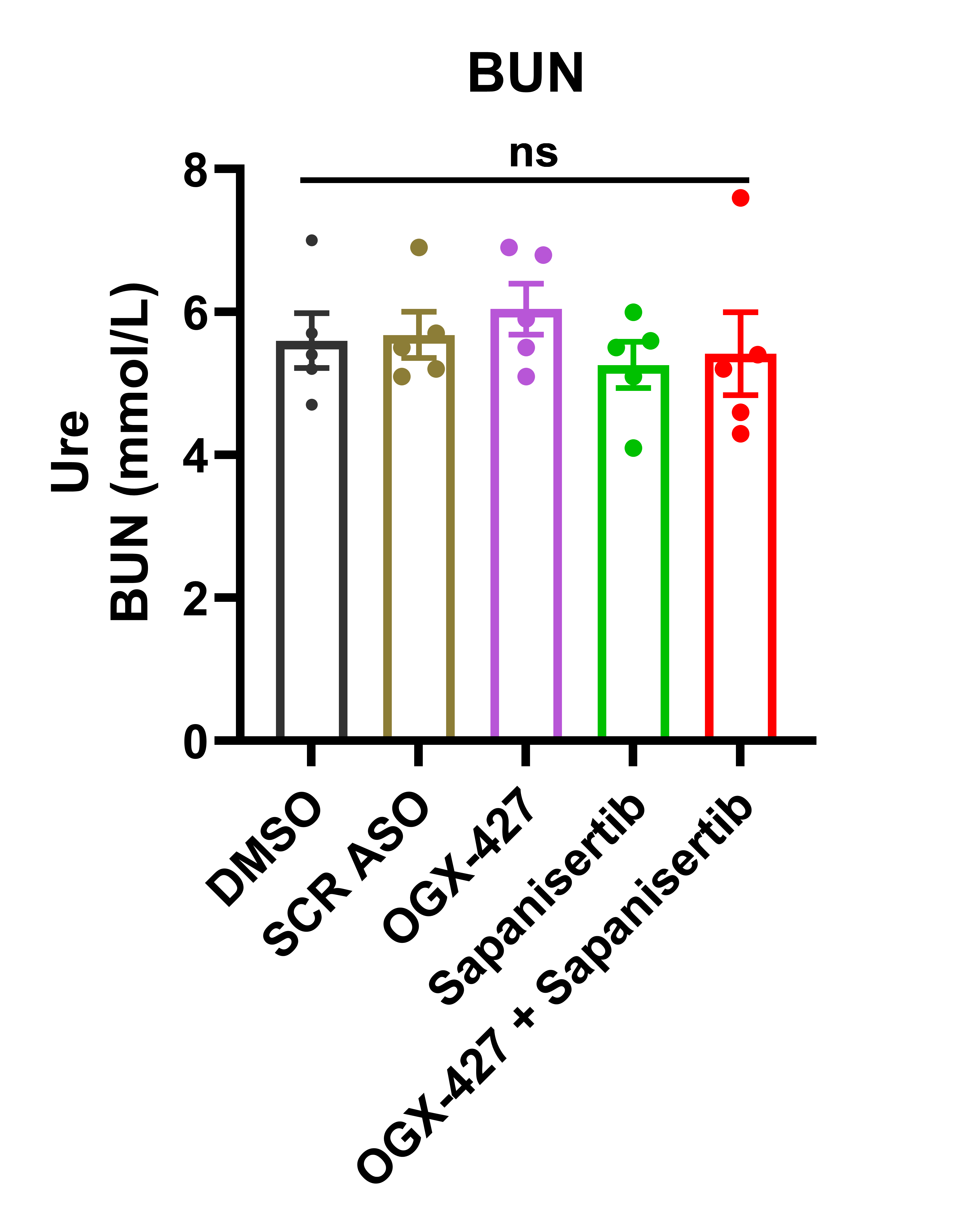

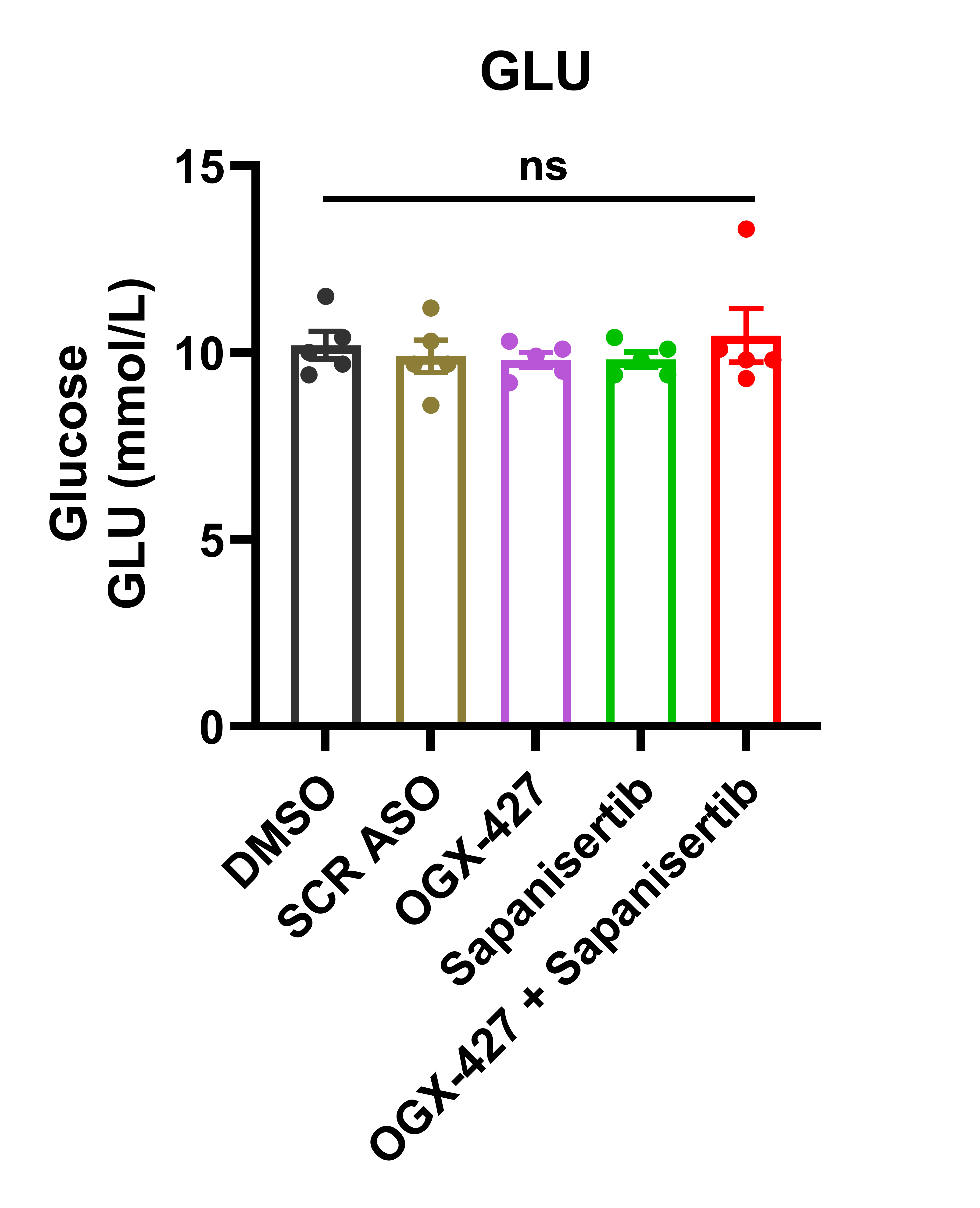

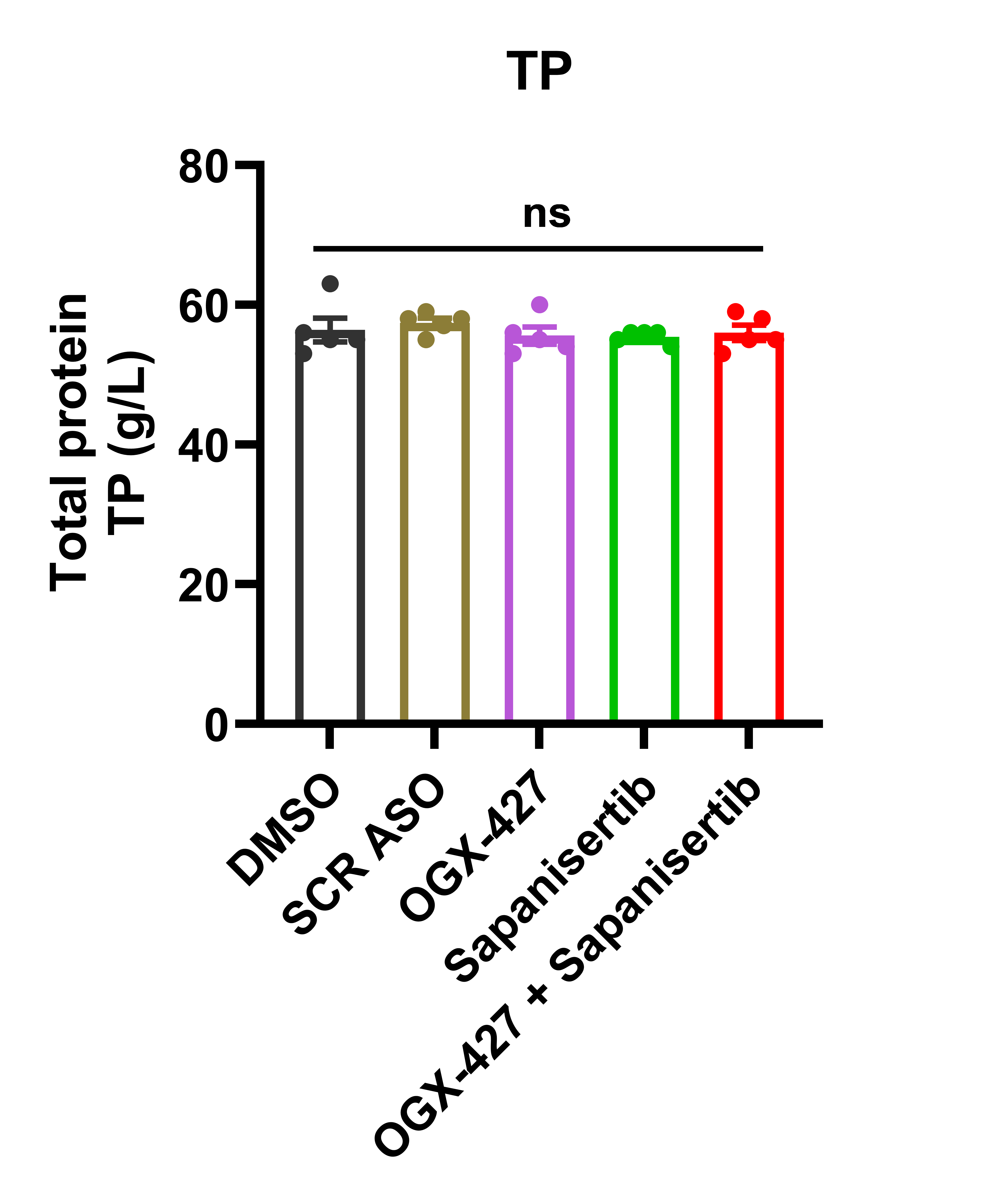


**Figure S5. H&E staining of major organs.** Liver, kidney, and spleen from three mice per group were examined for histopathological changes. No tissue damage or abnormalities were detected, indicating that the combination treatment is non-toxic compared with monotherapy or controls. Representative images are shown at 20× magnification.

**
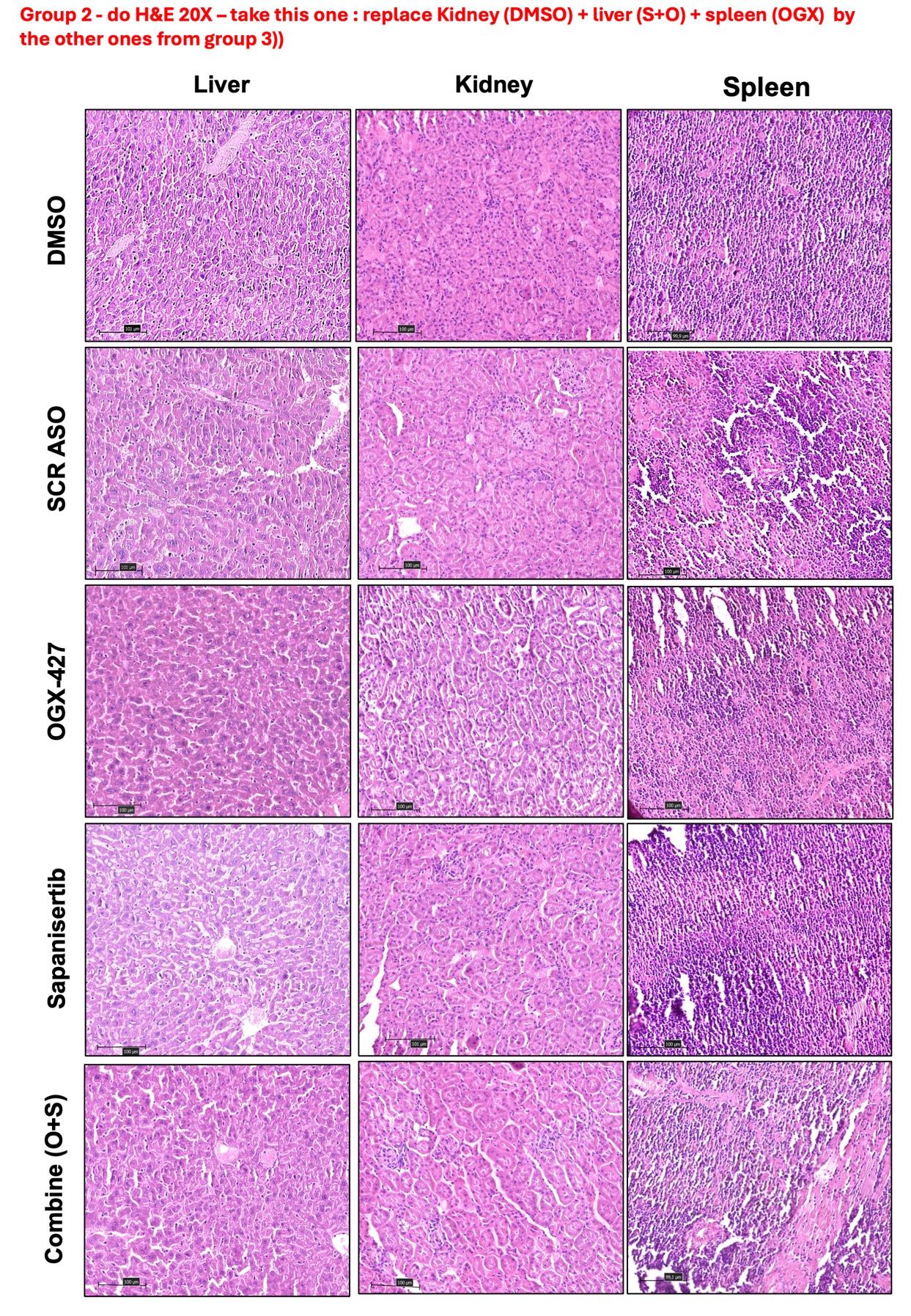
**

**Table S1. List of antibodies used in the study**

| **Target** | **Clone/**  **reference** | **Source** | **Company** | **Dilution** |
| --- | --- | --- | --- | --- |
| HSP27 | ADI-SPA-803 | Rabbit | Enzo Life science | WB: 1 : 20.000  IP:10μg/1ml lysate |
| Phosphorylated HSP27 (Ser78) | ADI-SPA-523 | Rabbit | Enzo Life science | WB: 1 : 500 |
| mTOR | #2983 (7C10) | Rabbit | Cell signaling | WB: 1 : 1000 |
| Phosphorylated mTOR (Ser2448) | #2971 | Rabbit | Cell signaling | WB: 1 : 1000 |
| Phosphorylated mTOR (Ser2481) | #2974 | Rabbit | Cell signaling | WB: 1: 1000 |
| RICTOR | ab70374 | Rabbit | abcam | WB: 1 : 1000 |
| RAPTOR | ab40768 | Rabbit | abcam | WB: 1 : 1000 |
| RAPTOR | #2280 (24C12) | Rabbit | Cell signaling | IP: 1 : 50 |
| p70S6K1 | #9202 | Rabbit | Cell signaling | WB: 1 : 1000 |
| Phosphorylated p70S6K1 (Thr389) | #9205 | Rabbit | Cell signaling | WB: 1 : 1000 |
| 4E-BP1 | #9452 | Rabbit | Cell signaling | WB: 1 : 1000 |
| Phosphorylated 4E-BP1 (Ser65) | #9451 | Rabbit | Cell signaling | WB: 1 : 1000 |
| PARP | #9542 | Rabbit | Cell signaing | WB: 1 : 1000 |
| GAPDH | sc-47724 | Mouse | Santa Cruz Biotechnology, Inc | WB: 1 : 3000 |
| Vinculin | ab129002 | Rabbit | abcam | WB: 1 : 2000 |
| Ki67 | 27309-1-AP | Rabbit | Proteintech | IF: 1 : 200 |
| Phosphorylated mTOR (Ser2448) | 67778-1-Ig | Rabbit | Proteintech | IF: 1 : 200 |
| CK5 | 27309-1-AP | Mouse | Proteintech | IF: 1 : 200 |
| CK8 | 17514-1-AP | Rabbit | Proteintech | IF: 1 : 200 |
| AR | 5153 | Rabbit | Cell signaling | IF: 1 : 200 |
| PSMA | NBP1-45057 | Mouse | Novus/Biotec | IF: 1 : 200 |
| Cleaved Caspase-3 (Asp175) | #9661 | Rabbit | Cell signaling | IHC: 1 : 200 |

**Table S2. List of primers and probes (qRT-PCR) used in the study**

| **Gene** | **Sequence** |
| --- | --- |
| **P70S6K1** | S6K1 Fw: CGACAGCCCAGATGACTCAA  S6K1 Rv: ATTTGACTGGGCTGACAGGT  Probe: CCTCGAAGATTTATTGGCAGCCCACG |
| **4E-BP1** | 4E-BP1 Fw: CCGGGAGGTACCAGGATCA  4E-BP1 Rv: CCCGCTTATCTTCTGGGCTA  Probe: CCAGCCAGAGCCACCTGCGC |
| **HSP27** | Hsp27 Fw: GAGATCACCGGCAAGCAC  Hsp27 Rv: ACAGGGAGGAGGAAACTTGG  Probe: AGCCATGCTCGTCCTGCCGC |
| **RAPTOR** | RAPTOR Fw: CCCGTCATGAAGATCCCAGA  Raptor Rv: TGCCCTTCTGAATGACTTGC  Probe: CGCCACTCCCGCTCCTTGCG |
