# Supplementary material for: HSP27 functional switch drives castration-resistant prostate cancer via mTOR pathway activation, highlighting promising combination therapies
Source: J Exp Clin Cancer Res. 2026 Mar 25;45:98. doi: 10.1186/s13046-026-03695-6 (PMC13085302; doi:10.1186/s13046-026-03695-6)

Figure 2 B

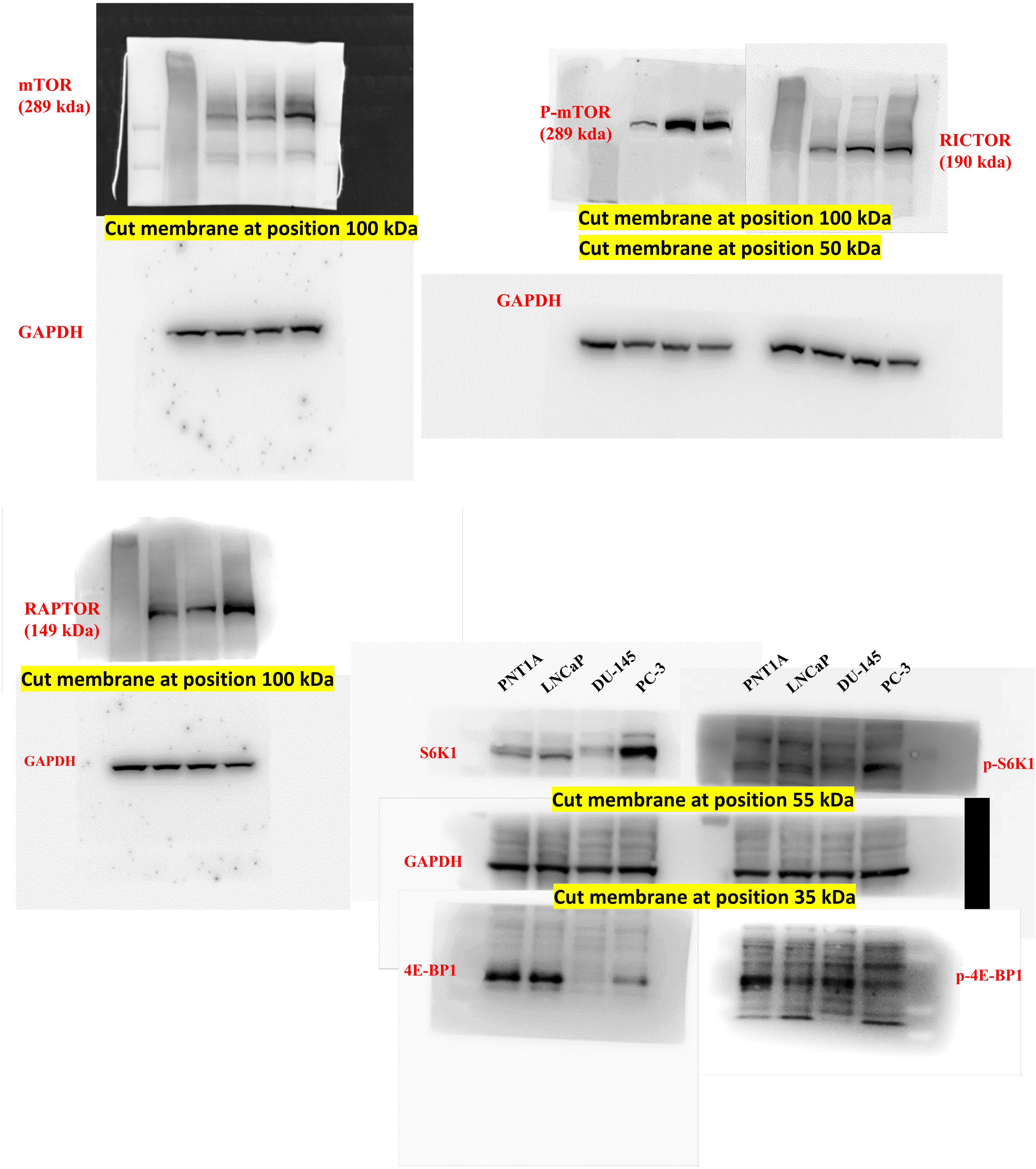

mTOR
(289 kda)
Cut membrane at position 100 kDa
GAPDH
P-mTOR
(289 kda)
RICTOR
(190 kda)
Cut membrane at position 100 kDa
Cut membrane at position 50 kDa
GAPDH
RAPTOR
(149 kDa)
Cut membrane at position 100 kDa
GAPDH
PNT1A
LNCaP
DU-145
PC-3
PNT1A
LNCaP
DU-145
PC-3
S6K1
p-S6K1
Cut membrane at position 55 kDa
GAPDH
Cut membrane at position 35 kDa
4E-BP1
p-4E-BP1

Figure 2 C, D

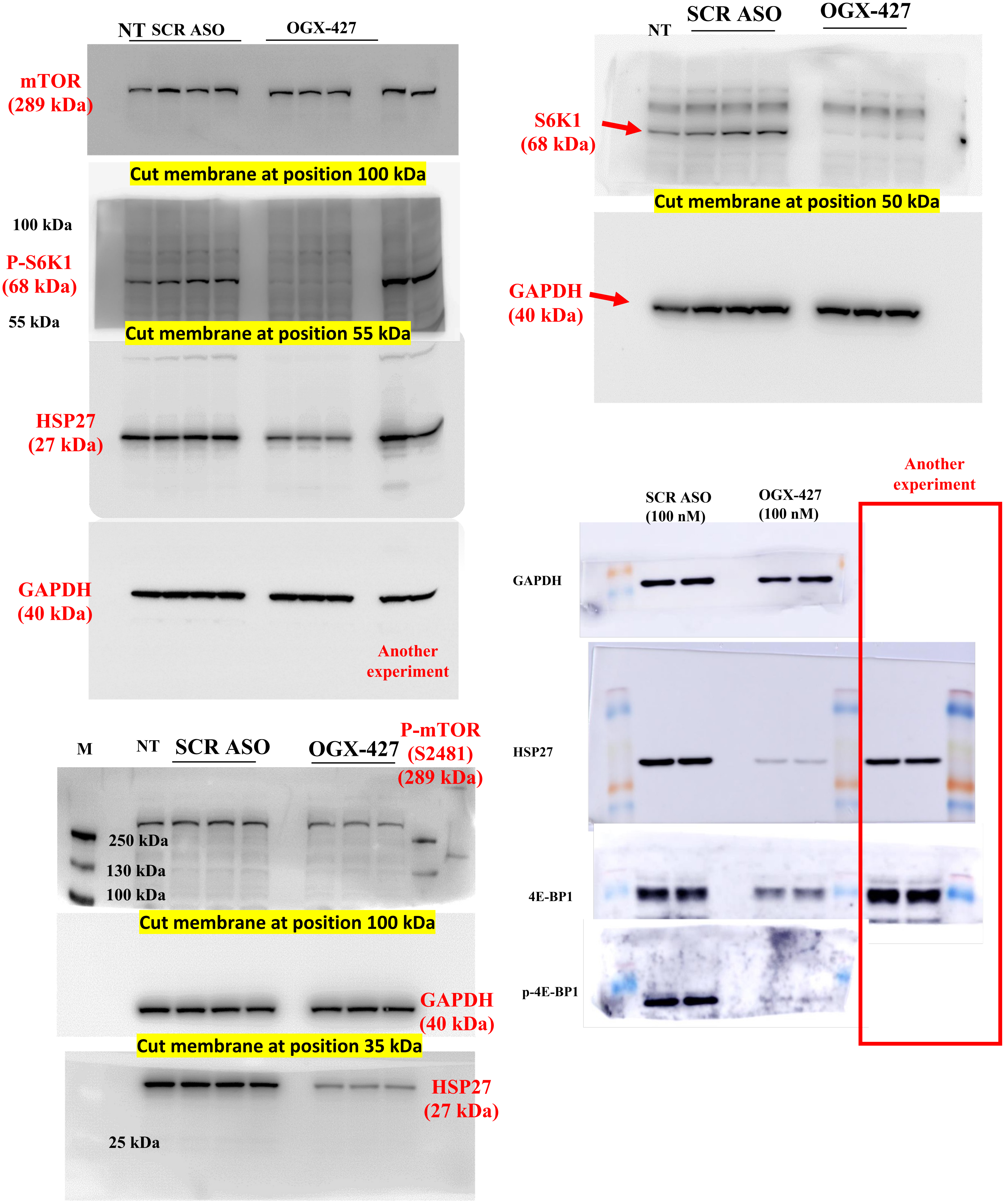

NT SCR ASO
OGX-427
mTOR (289 kDa)
Cut membrane at position 100 kDa
100 kDa
P-S6K1 (68 kDa)
55 kDa
Cut membrane at position 55 kDa
HSP27 (27 kDa)
GAPDH (40 kDa)
Another experiment
NT
SCR ASO
OGX-427
S6K1 (68 kDa)
Cut membrane at position 50 kDa
GAPDH (40 kDa)
Another experiment
SCR ASO (100 nM)
OGX-427 (100 nM)
GAPDH
HSP27
4E-BP1
p-4E-BP1
M
NT
SCR ASO
OGX-427
P-mTOR (S2481) (289 kDa)
250 kDa
130 kDa
100 kDa
Cut membrane at position 100 kDa
GAPDH (40 kDa)
Cut membrane at position 35 kDa
HSP27 (27 kDa)
25 kDa

Figure 2 C

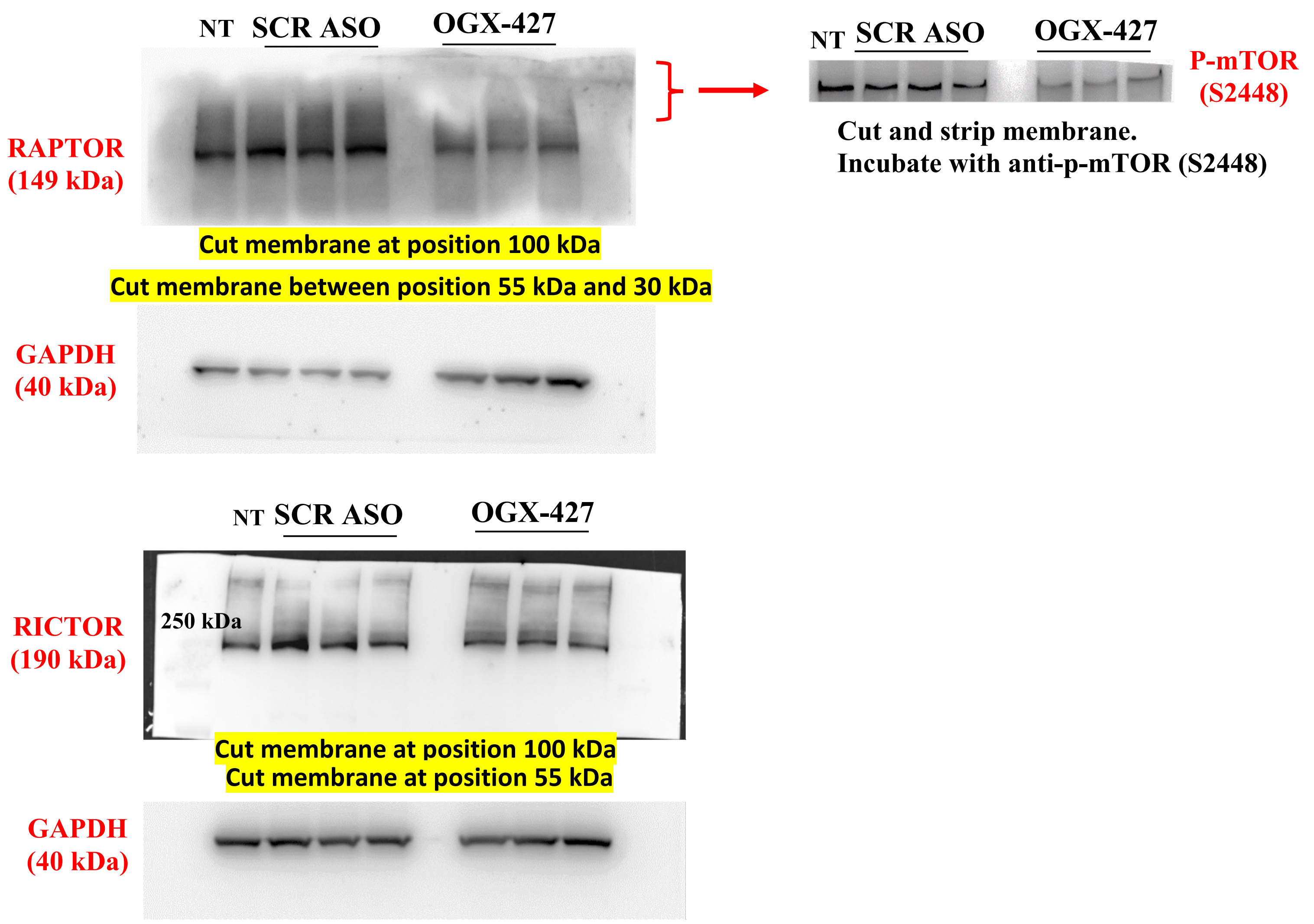
NT SCR ASO OGX-427
RAPTOR (149 kDa)
Cut membrane at position 100 kDa
Cut membrane between position 55 kDa and 30 kDa
GAPDH (40 kDa)
NT SCR ASO OGX-427
P-mTOR (S2448)
Cut and strip membrane.
Incubate with anti-p-mTOR (S2448)
NT SCR ASO OGX-427
RICTOR (190 kDa)
250 kDa
Cut membrane at position 100 kDa
Cut membrane at position 55 kDa
GAPDH (40 kDa)

Figure 2 E

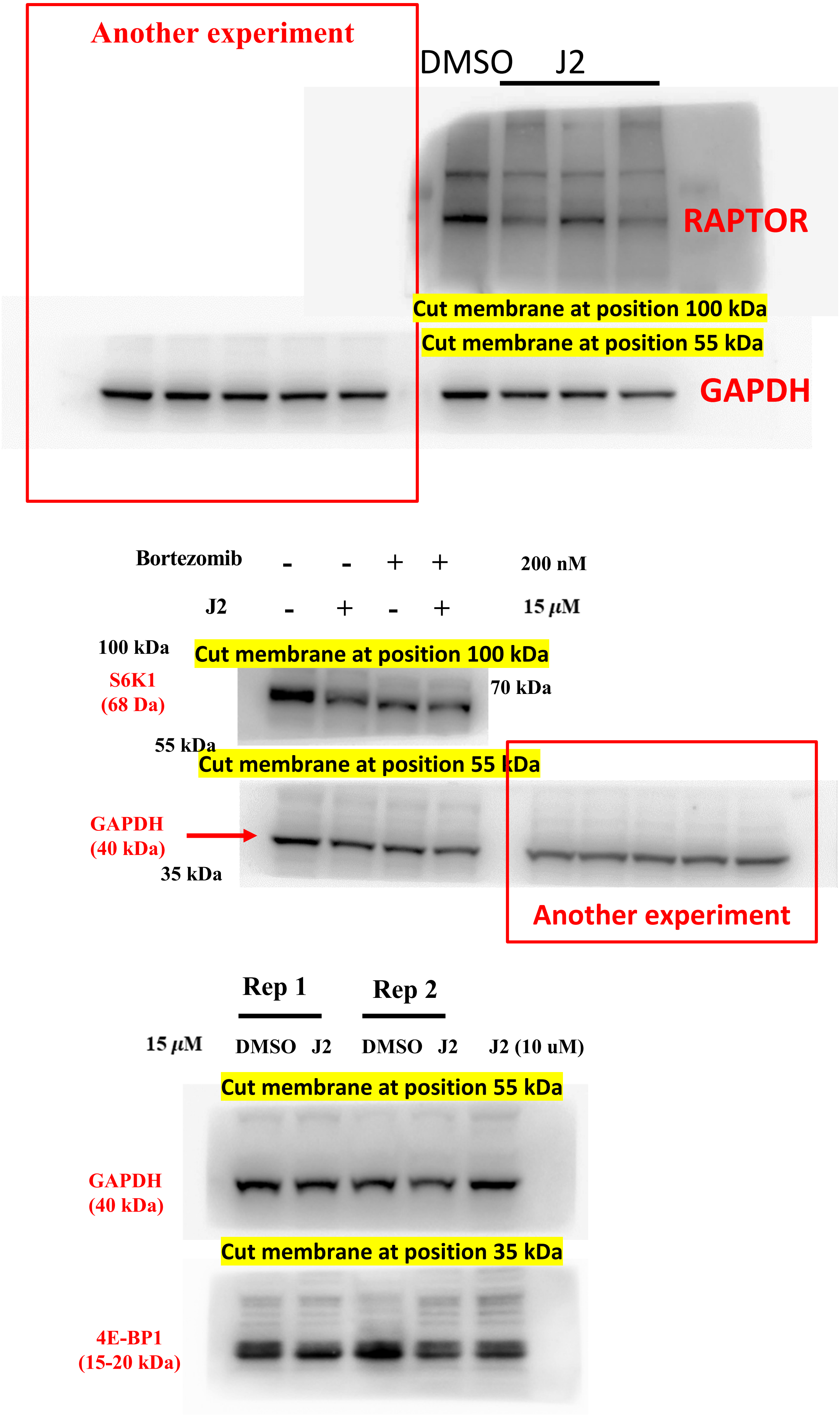

Another experiment
DMSO
J2
RAPTOR
Cut membrane at position 100 kDa
Cut membrane at position 55 kDa
GAPDH
Bortezomib
-
-
+
+
200 nM
J2
-
+
-
+
15 μM
100 kDa
Cut membrane at position 100 kDa
S6K1
(68 Da)
70 kDa
55 kDa
Cut membrane at position 55 kDa
GAPDH
(40 kDa)
35 kDa
Another experiment
Rep 1
Rep 2
15 μM
DMSO
J2
DMSO
J2
J2 (10 uM)
Cut membrane at position 55 kDa
GAPDH
(40 kDa)
Cut membrane at position 35 kDa
4E-BP1
(15-20 kDa)

Figure 3A

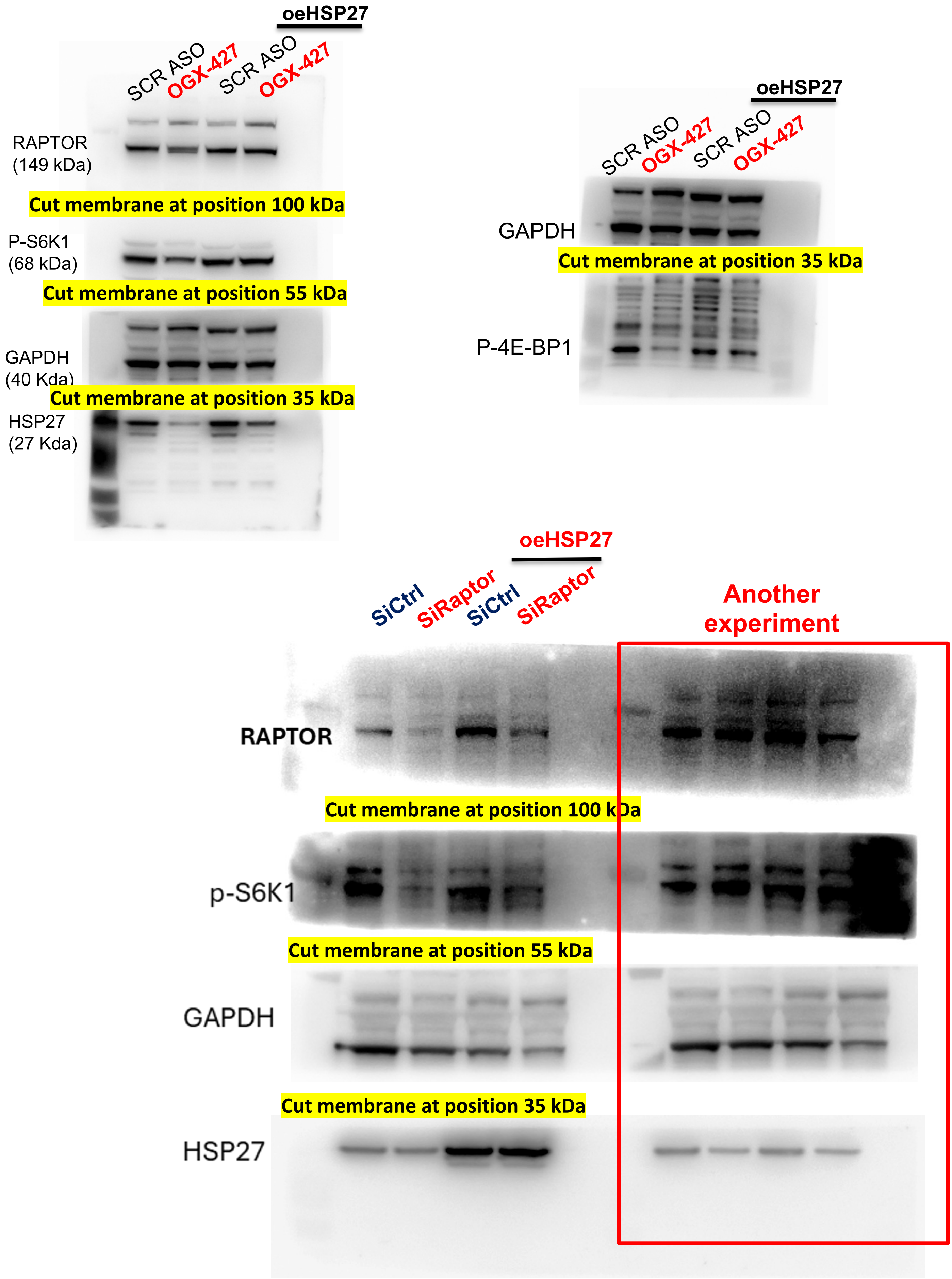
oeHSP27
SCR ASO
OGX-427
SCR ASO
OGX-427
RAPTOR (149 kDa)
Cut membrane at position 100 kDa
P-S6K1 (68 kDa)
Cut membrane at position 55 kDa
GAPDH (40 Kda)
Cut membrane at position 35 kDa
HSP27 (27 Kda)
oeHSP27
SCR ASO
OGX-427
SCR ASO
OGX-427
GAPDH
Cut membrane at position 35 kDa
P-4E-BP1
oeHSP27
SiCtrl
SiRaptor
SiCtrl
SiRaptor
Another experiment
RAPTOR
Cut membrane at position 100 kDa
p-S6K1
Cut membrane at position 55 kDa
GAPDH
Cut membrane at position 35 kDa
HSP27

Figure 3B

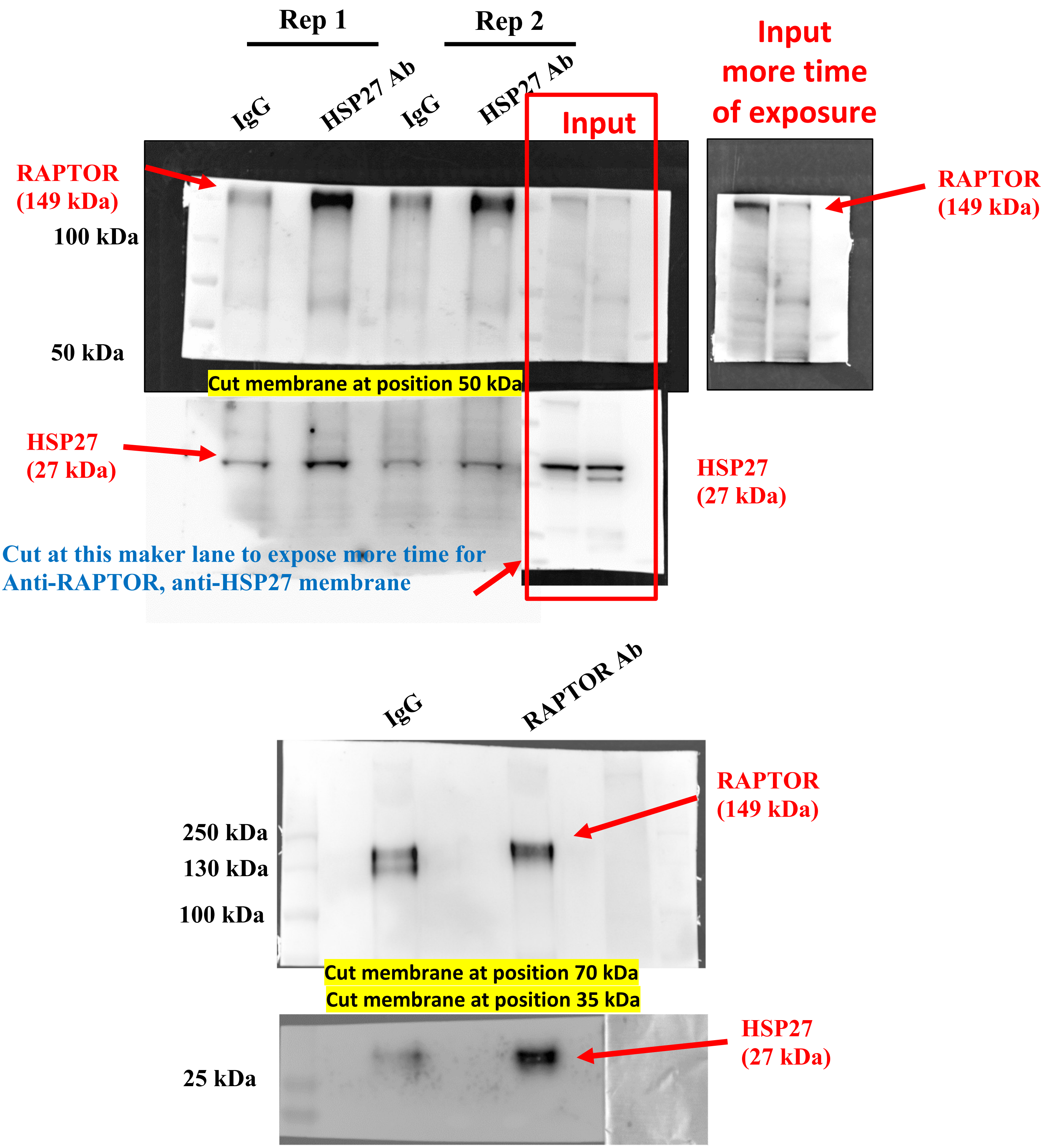

Rep 1
Rep 2
IgG
HSP27 Ab
IgG
HSP27 Ab
Input
Input
more time
of exposure
RAPTOR
(149 kDa)
100 kDa
50 kDa
RAPTOR
(149 kDa)
Cut membrane at position 50 kDa
HSP27
(27 kDa)
HSP27
(27 kDa)
Cut at this maker lane to expose more time for
Anti-RAPTOR, anti-HSP27 membrane
IgG
RAPTOR Ab
250 kDa
130 kDa
100 kDa
RAPTOR
(149 kDa)
Cut membrane at position 70 kDa
Cut membrane at position 35 kDa
25 kDa
HSP27
(27 kDa)

Figure 3C

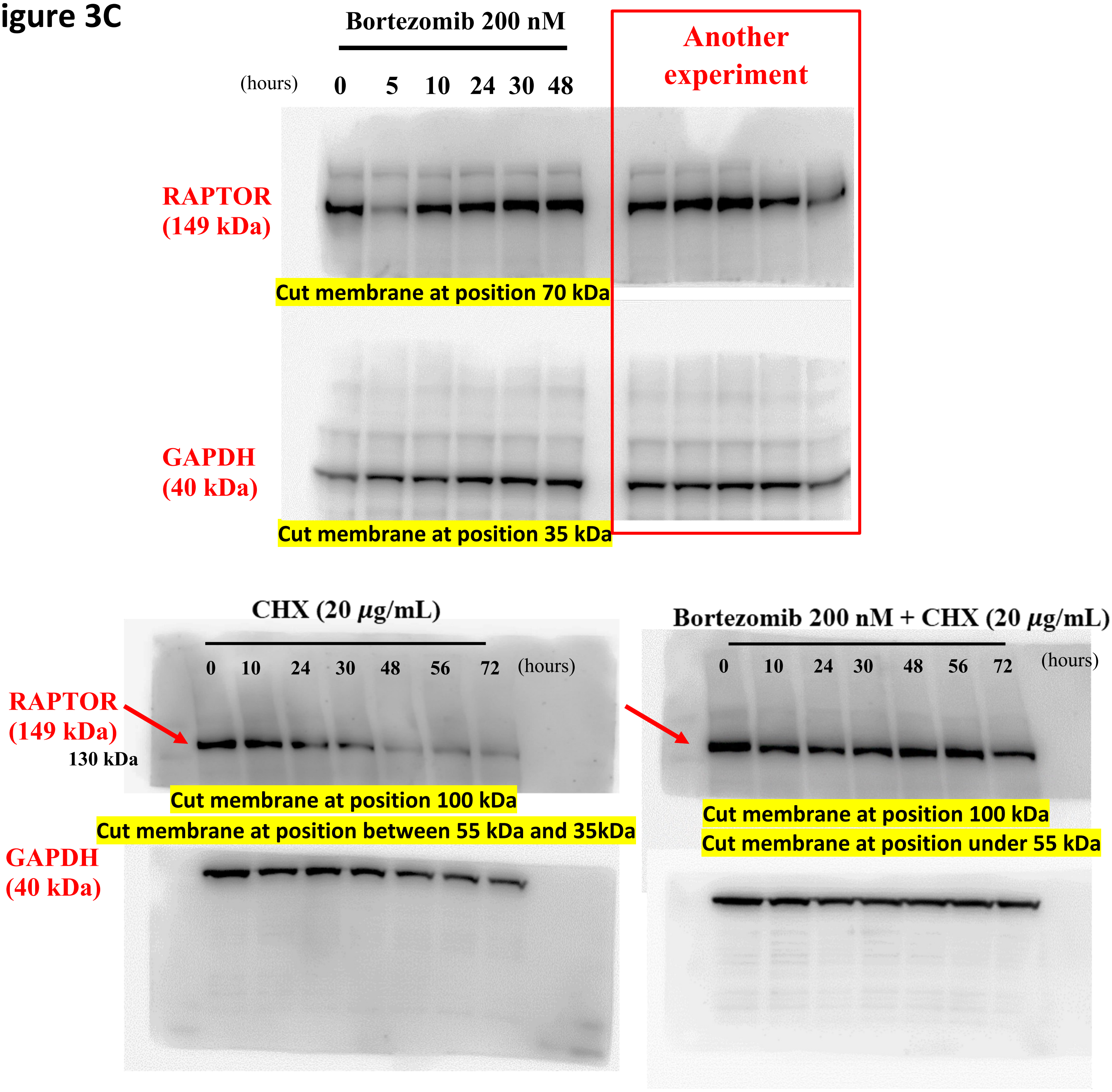

Bortezomib 200 nM
(hours) 0 5 10 24 30 48
Another experiment
RAPTOR (149 kDa)
Cut membrane at position 70 kDa
GAPDH (40 kDa)
Cut membrane at position 35 kDa
CHX (20 μg/mL)
0 10 24 30 48 56 72 (hours)
RAPTOR (149 kDa)
130 kDa
Cut membrane at position 100 kDa
Cut membrane at position between 55 kDa and 35kDa
GAPDH (40 kDa)
Bortezomib 200 nM + CHX (20 μg/mL)
0 10 24 30 48 56 72 (hours)
Cut membrane at position 100 kDa
Cut membrane at position under 55 kDa

Figure 3D

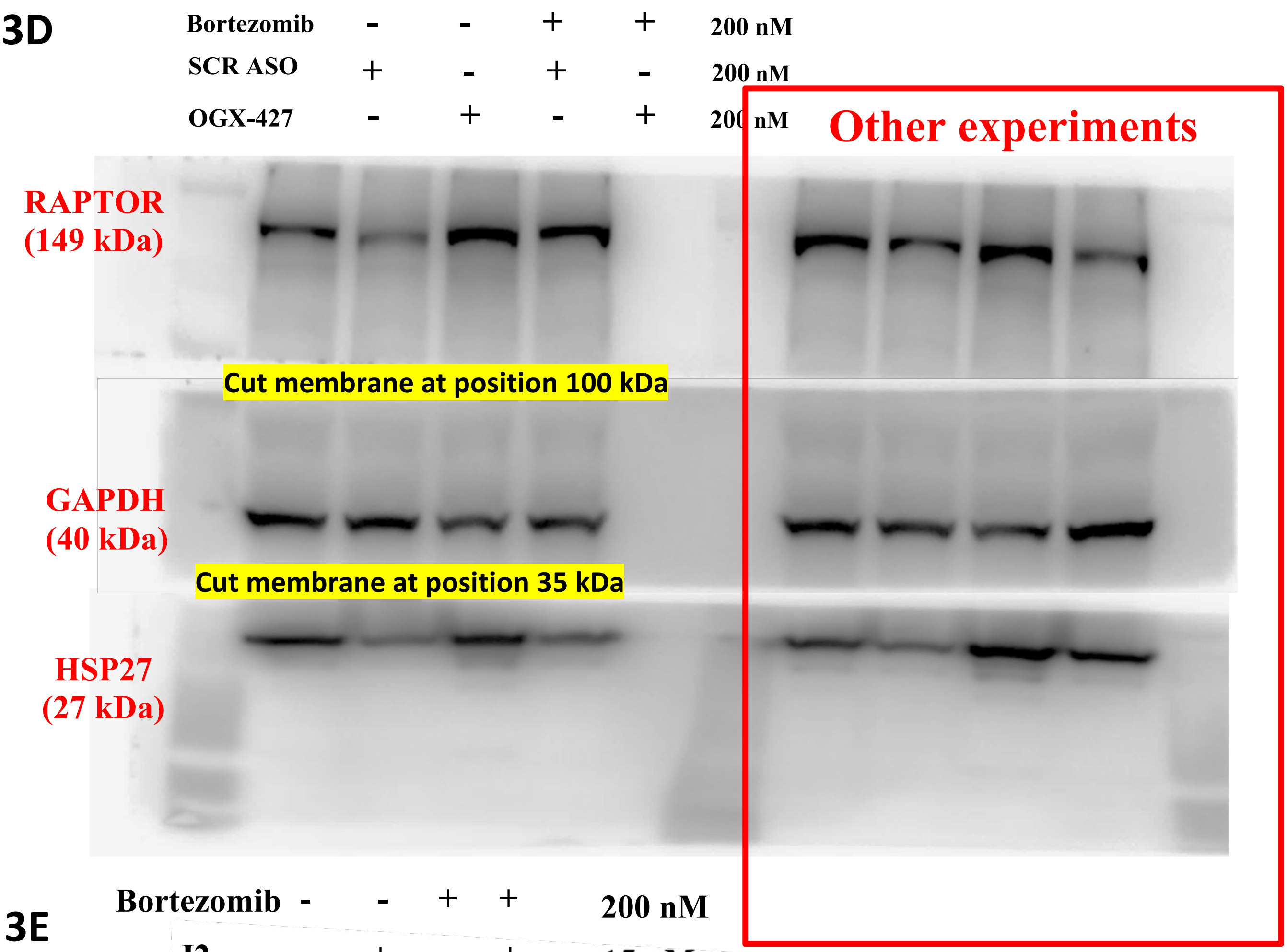

Figure 3E

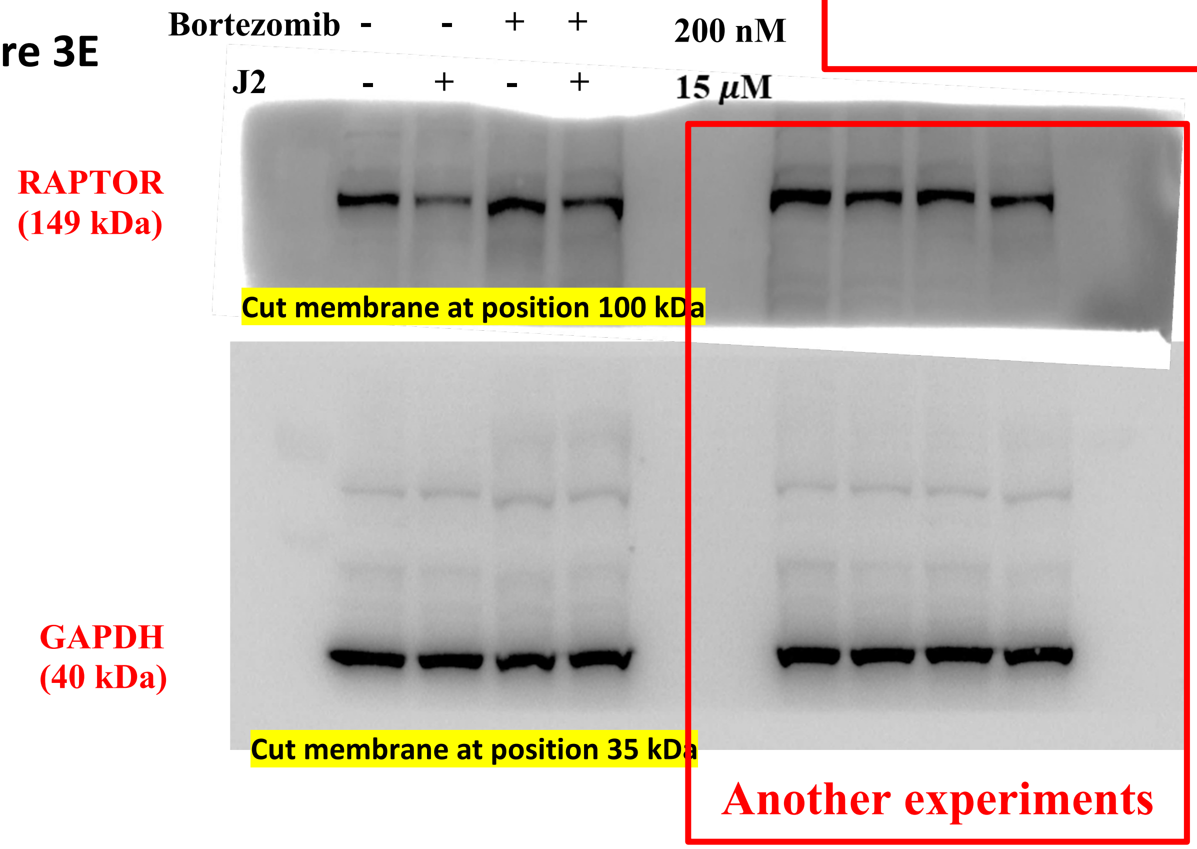

Figure 4

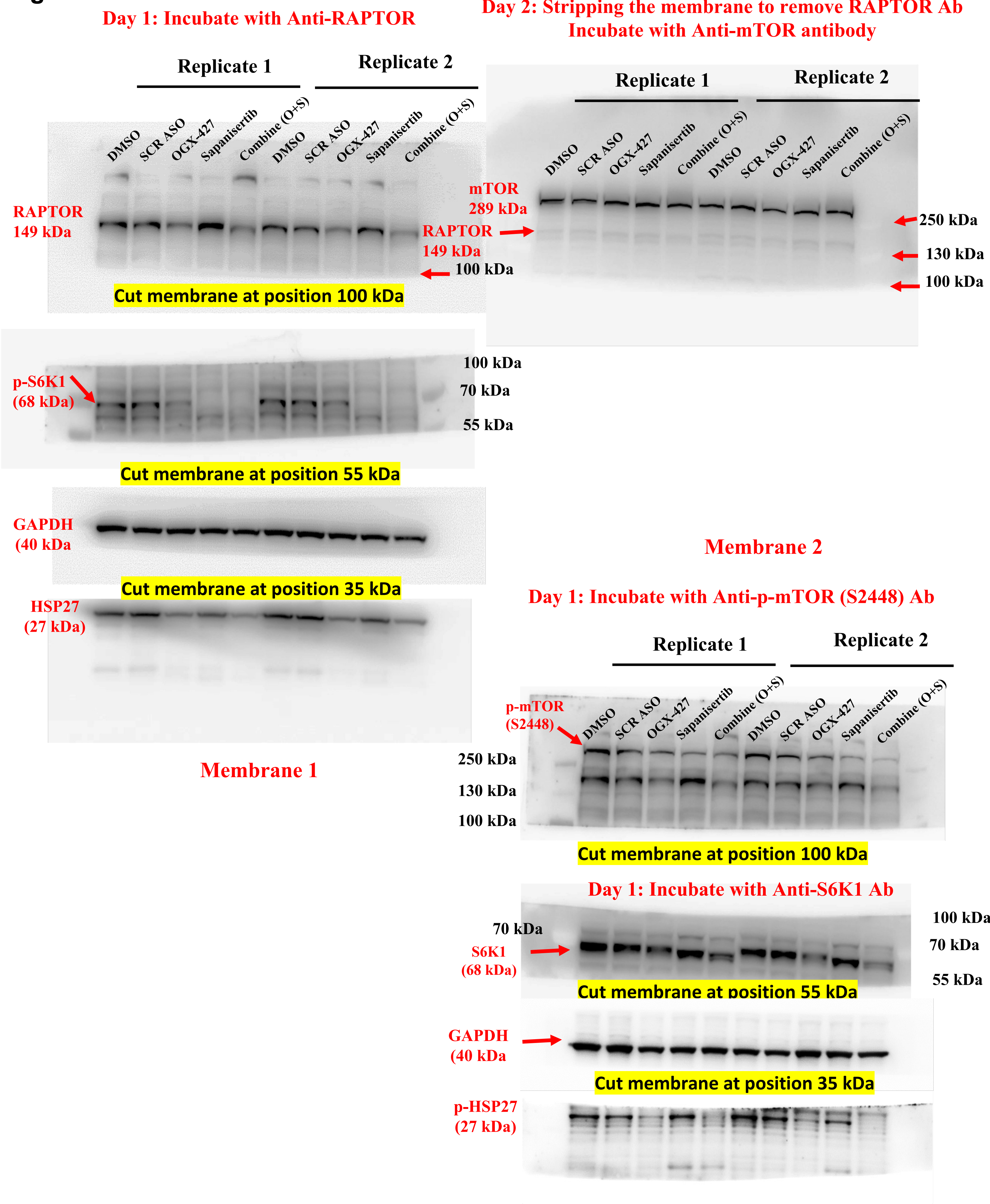
Day 1: Incubate with Anti-RAPTOR
Replicate 1
Replicate 2
DMSO
SCR ASO
OGX-427
Sapanisertib
Combine (O+S)
RAPTOR
149 kDa
100 kDa
Cut membrane at position 100 kDa
p-S6K1
(68 kDa)
100 kDa
70 kDa
55 kDa
Cut membrane at position 55 kDa
GAPDH
(40 kDa
Cut membrane at position 35 kDa
HSP27
(27 kDa)
Membrane 1
Day 2: Stripping the membrane to remove RAPTOR Ab
Incubate with Anti-mTOR antibody
mTOR
289 kDa
RAPTOR
149 kDa
250 kDa
130 kDa
100 kDa
Membrane 2
Day 1: Incubate with Anti-p-mTOR (S2448) Ab
p-mTOR
(S2448)
250 kDa
130 kDa
100 kDa
Cut membrane at position 100 kDa
Day 1: Incubate with Anti-S6K1 Ab
70 kDa
S6K1
(68 kDa)
100 kDa
70 kDa
55 kDa
Cut membrane at position 55 kDa
GAPDH
(40 kDa
Cut membrane at position 35 kDa
p-HSP27
(27 kDa)

Figure 5: Original blots

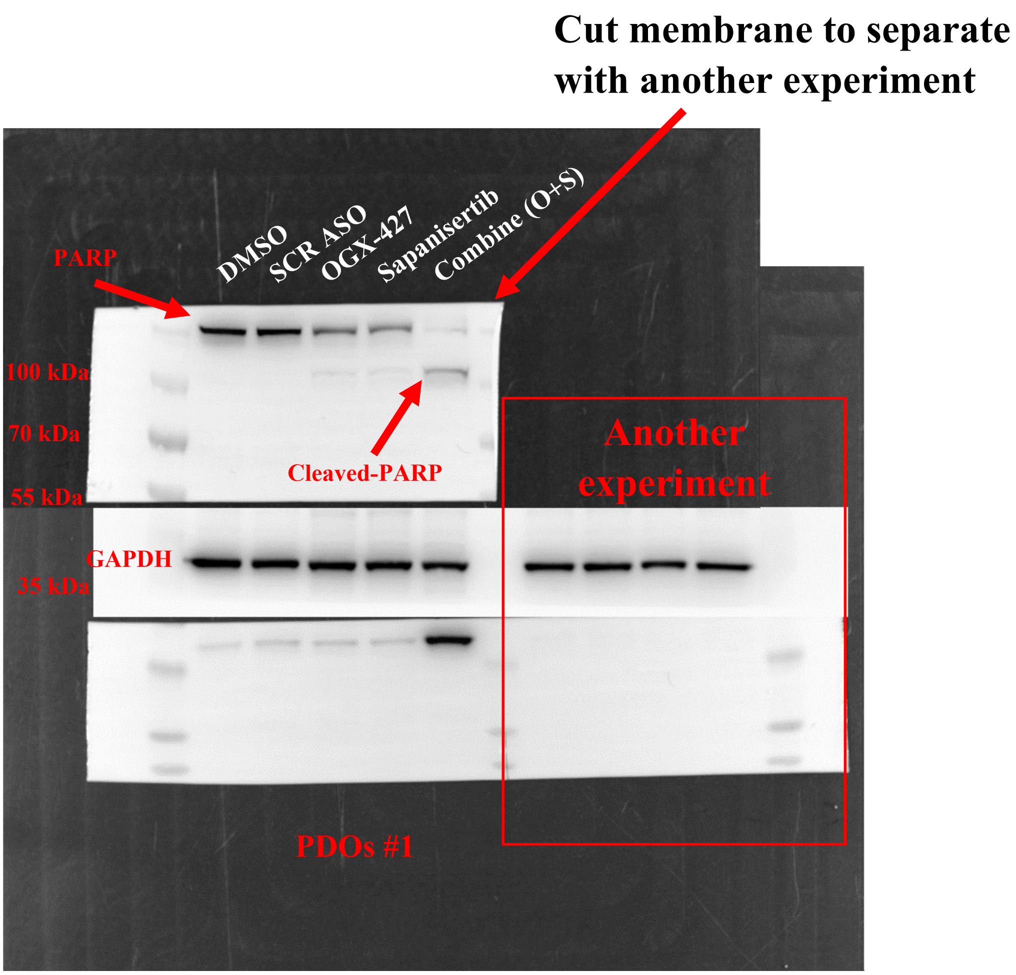

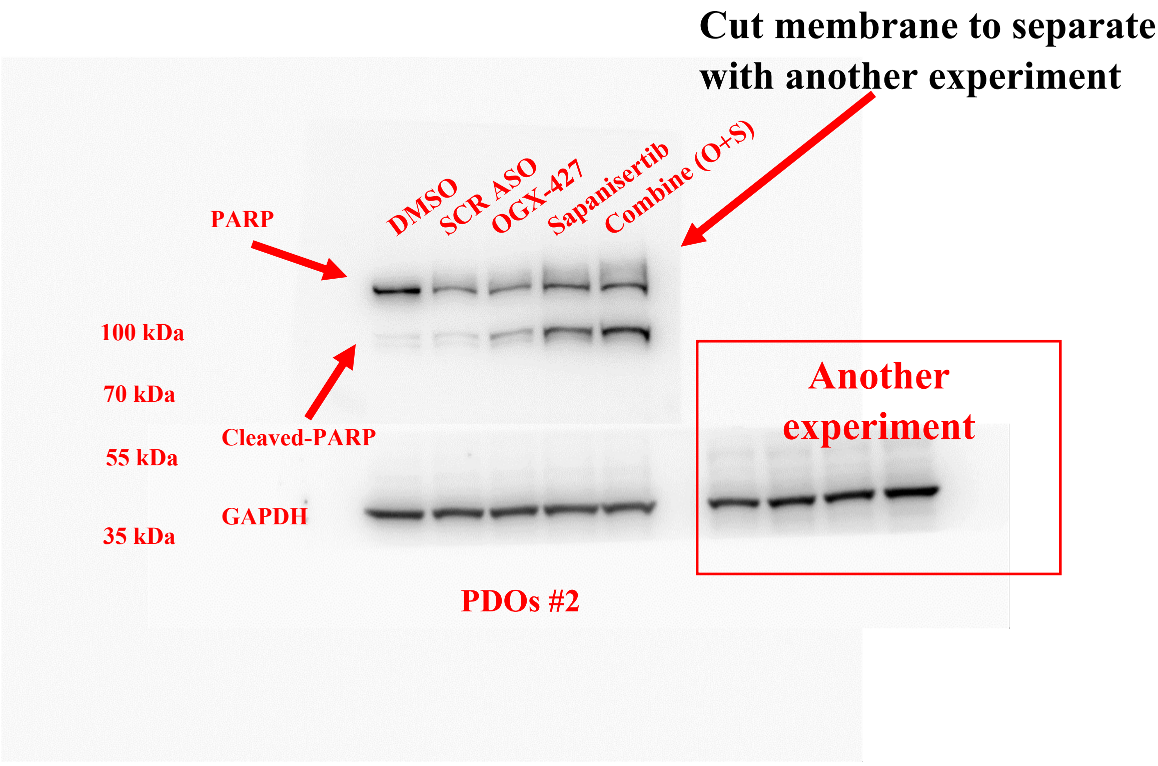

Figure 6: In vivo

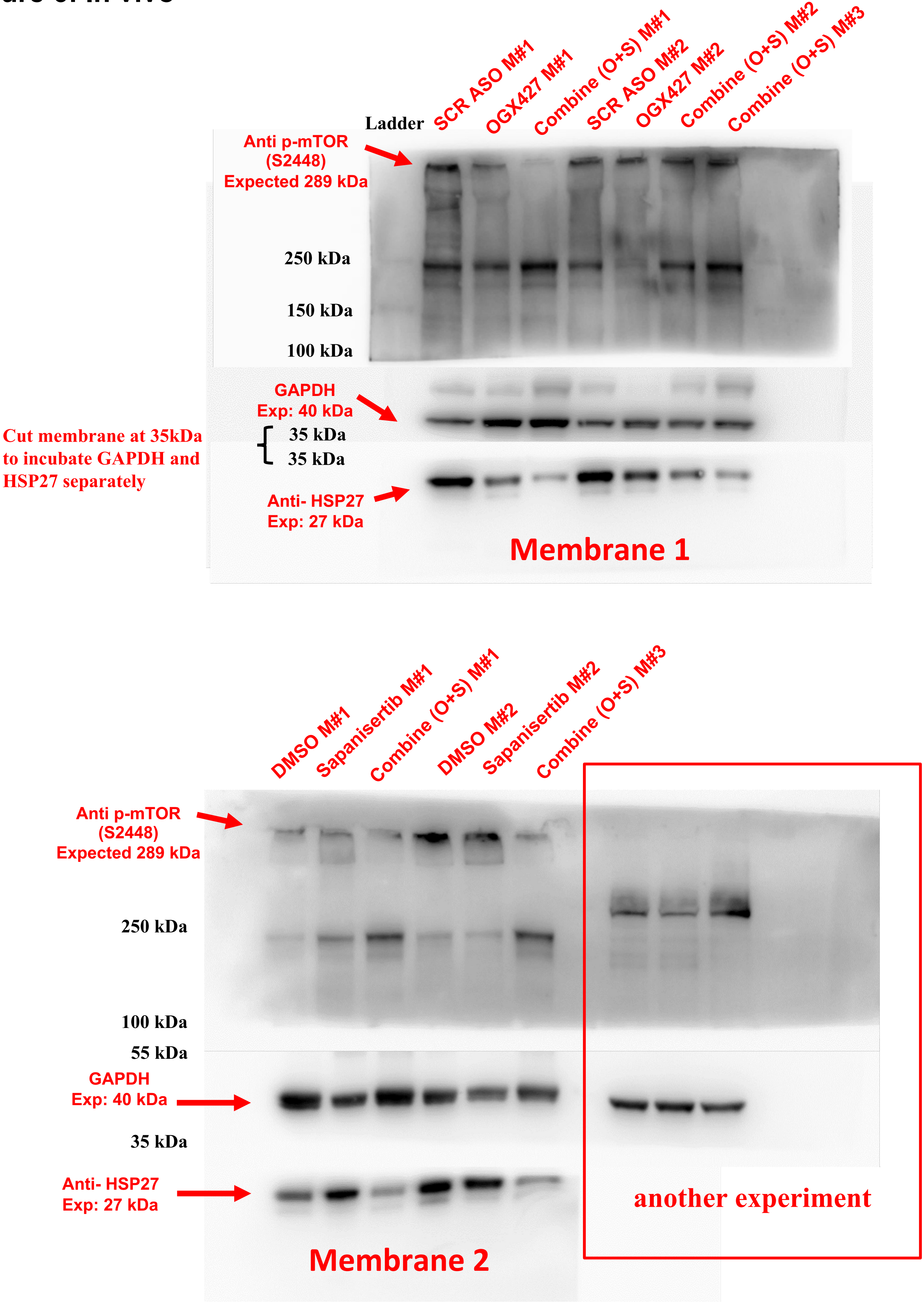

Supplement: Supplementary file 4 — Supplementary Material 4. [file 13046_2026_3695_MOESM4_ESM.pdf]
